# Structures of the Crimean-Congo hemorrhagic fever virus RNA-dependent RNA polymerase

**DOI:** 10.1038/s41421-026-00905-5

**Published:** 2026-07-14

**Authors:** Mengyun Li, Kaixiang Zhu, Yanan Liu, Kun Shang, Yuanhao Li, Xinyue Wang, Jianing Wang, Jie Jia, Xin Ai, Dongcun Ni, Sheng Cui, Hongtao Zhu, Xiaopan Gao

**Affiliations:** 1 NHC Key Laboratory of Systems Biology of Pathogens, National Institute of Pathogen Biology, Chinese Academy of Medical Sciences & Peking Union Medical College, Beijing, China; 2 Key Laboratory of Pathogen Infection Prevention and Control (Ministry of Education), National Institute of Pathogen Biology, Chinese Academy of Medical Sciences & Peking Union Medical College, Beijing, China; 3 State Key Laboratory of Respiratory Health and Multimorbidity, National Institute of Pathogen Biology, Chinese Academy of Medical Sciences & Peking Union Medical College, Beijing, China; 4 Beijing National Laboratory for Condensed Matter Physics, Institute of Physics, Chinese Academy of Sciences, Beijing, China; 5 University of Chinese Academy of Sciences, Beijing, China; 6 Songshan Lake Materials Laboratory, Dongguan, Guangdong China; 7 Medical School, Yan’an University, Yan’an, Shaanxi China; 8 School of Engineering, University of Warwick, Coventry, West Midlands UK; 9 School of Pharmacy, International Cancer Center, Guangdong Key Laboratory of Genome Instability and Human Disease Prevention, Shenzhen University Medical School, Shenzhen, Guangdong China

**Keywords:** Molecular biology, Structural biology

## Abstract

Crimean-Congo hemorrhagic fever virus (CCHFV), designated by the WHO as a priority pathogen under its R&D Blueprint for emerging epidemics, poses a major global health threat, yet licensed vaccines or specific antiviral treatments are lacking. As the sole viral enzyme responsible for genome replication and transcription, the CCHFV L protein is a large, multienzymatic protein, but its exceptional size (> 450 kDa) and extensive domain architecture have hindered structural analysis. Here, we present high-resolution cryo-electron microscopy structures of the full-length CCHFV L protein in its apo state and bound to the 5′ viral RNA promoter. These structures reveal the largest polymerase known among *Bunyavirales* and demonstrate that the apo form adopts a highly flexible conformation in which multiple functional elements remain disordered. Binding of the 5′ promoter RNA triggers extensive conformational rearrangements that organize these elements into a catalytically competent active site. We define a conserved 5′ hook-binding mode and identify two previously unrecognized residues (K1545 and E1637) that form a constriction at the NTP entry channel, representing newly defined regulatory motifs J and K conserved across *Bunyavirales*. We further characterize an expanded pendant domain unique to nairoviruses that, although not essential for promoter binding, likely modulates template movement within the internal tunnel during RNA synthesis. Our results provide the first structural framework for a nairovirus polymerase, illuminate the mechanisms of CCHFV RNA synthesis, and establish a foundation for structure-guided antiviral development against this high-priority pathogen.

## Introduction

Crimean-Congo hemorrhagic fever virus (CCHFV), a segmented negative-sense RNA virus (sNSV) of the genus *Orthonairovirus* within the family *Nairoviridae* (order *Bunyavirales*)^1^, is among the most important tick-borne zoonotic pathogens and the causative agent of Crimean-Congo hemorrhagic fever. The virus is maintained primarily in nature through ticks of the genus *Hyalomma*, which serve as both vectors and reservoirs. Consequently, the geographic distribution of CCHFV closely parallels that of these ticks, whose habitats are expanding under the influence of climate change, migratory birds, and livestock trade, thereby increasing the risk of viral spread^2,3^. To date, CCHFV has been reported in more than 30 countries across Africa, Asia, Europe, and the Middle East^4–7^. The virus has an unusually broad host range, infecting a variety of domestic and wild animals as well as humans. Human infection occurs through the bite of infected ticks, contact with viremic livestock or wildlife, and, occasionally, direct person-to-person transmission via exposure to infectious bodily fluids^4^. Clinically, CCHFV infection can lead to severe hemorrhagic disease, with reported case fatality rates ranging from 10% to 40%^8–10^. Although substantial progress has been made in understanding viral pathogenesis and in testing candidate vaccines and antivirals, there are still no licensed vaccines or specific therapeutic agents available for CCHFV infection. Reflecting its wide distribution, expanding ecological range, high mortality, epidemic potential, and lack of effective countermeasures, CCHFV has been listed by the WHO R&D Blueprint as a priority pathogen^11,12^.

CCHFV possesses a tri-segmented, negative-sense RNA genome, comprising the small (S), medium (M), and large (L) segments^4^. The S segment encodes the nucleoprotein (NP), which encapsulates the viral RNA; the M segment encodes the glycoprotein precursor (GPC), which is post-translationally processed into the envelope glycoproteins Gn and Gc; and the L segment encodes a large multifunctional L protein that contains the RNA-dependent RNA polymerase (RdRp) responsible for viral genome replication and transcription^4,13^. A distinctive feature of CCHFV and of nairoviruses in genera is the exceptional size and structural complexity of their L protein. In contrast to the L proteins of other bunyaviruses, which typically range from 240 kDa to 260 kDa, the nairovirus L protein can exceed 450 kDa because of the presence of additional extensions and multiple inserted accessory domains while retaining the overall architecture characteristics of sNSVs. Most notably, at the N-terminus of the CCHFV L protein lies an ovarian tumor (OTU)-like protease domain, a unique element absent from other sNSVs. This OTU domain exhibits deubiquitinase and deISGylase activities, enabling the virus to antagonize host innate immune signaling and evade antiviral responses^14^.

In common with other *Bunyavirales*, each viral genomic segment associates with multiple copies of the nucleoprotein (NP) and a single L protein, forming a ribonucleoprotein (RNP) complex that constitutes structural and functional units of viral genome replication and transcription in the host cell cytoplasm^15^. During replication, the L protein synthesizes full-length complementary RNA (cRNA) and genomic viral RNA (vRNA), which serve as templates for subsequent rounds of RNA synthesis^16–18^. During transcription, the polymerase produces capped viral mRNAs that are recognized by the host translation machinery to generate viral proteins. Transcription is typically initiated through a cap-snatching mechanism in which host-derived 5′ capped RNAs are bound by a cap-binding domain (CBD) and cleaved by an endonuclease (EN) domain located within the L protein several nucleotides downstream of the cap^19^. The resulting short capped RNA fragments then serve as primers for viral mRNA synthesis.

Given its central role in viral replication and transcription, the L protein represents a key antiviral target across sNSVs. Consequently, substantial research efforts have focused on elucidating the structures and mechanisms of viral RdRps to facilitate the rational design of broad-spectrum antivirals^16,20^. In recent years, high-resolution structures of L proteins, both in isolation and captured in functionally relevant catalytic states, have been determined from several members of the order sNSVs, including those of La Crosse virus (LACV, *Peribunyaviridae*)^21–23^, severe fever with thrombocytopenia syndrome virus (SFTSV, *Phenuiviridae*)^24–27^, Tomato spotted wilt orthotospovirus (TSWV, *Tospoviridae*)^28^, Hantaan virus and Sin Nombre virus (HTNV, SNV, *Hantaviridae*)^29–32^ and Rift Valley fever virus (RVFV, *Phenuiviridae*)^33^, as well as the arenaviruses Lassa virus (LASV) and Machupo virus (MACV)^34,35^. These structural studies have revealed both shared architectural principles and unique domain adaptations among bunyavirus polymerases, highlighting the remarkable flexibility and multifunctionality of this molecular machine. Despite these advances, the structure of the nairovirus L protein, including that in CCHFV, has remained elusive. The extraordinary molecular mass of the nairovirus L protein (over 450 kDa), combined with its highly intricate multi-domain organization and the presence of distinct structural insertions, has made the characterization of its expression, purification and high-resolution structure particularly challenging.

In this study, we established a robust system for the expression and purification of the full-length CCHFV L protein (~450 kDa). We report the high-resolution structures of the CCHFV L protein, both in isolation and in complex with the 5′ viral RNA, providing the first detailed insights into the organization of a polymerase from the *Nairoviridae* family and revealing molecular specificities that distinguish it from other *Bunyavirales* polymerases. Structural analysis revealed two highly conserved residues among sNSVs that are likely involved in nucleoside triphosphate (NTP) entry during RNA synthesis. In addition, we discovered a large and unique pendant insertion domain, an unprecedented structural element not observed in other sNSV polymerases. Overall, the CCHFV L protein exhibits a global architecture resembling that of influenza and bunyavirus polymerases but contains distinct local adaptations, including a family-specific insertion domain that may regulate enzymatic function. These findings significantly advance our understanding of the structural basis and regulatory mechanisms of RNA synthesis in sNSVs and provide a foundation for structure-guided antiviral drug design.

## Results

### Overall structure of the CCHFV L Core

**Fig. 1:**
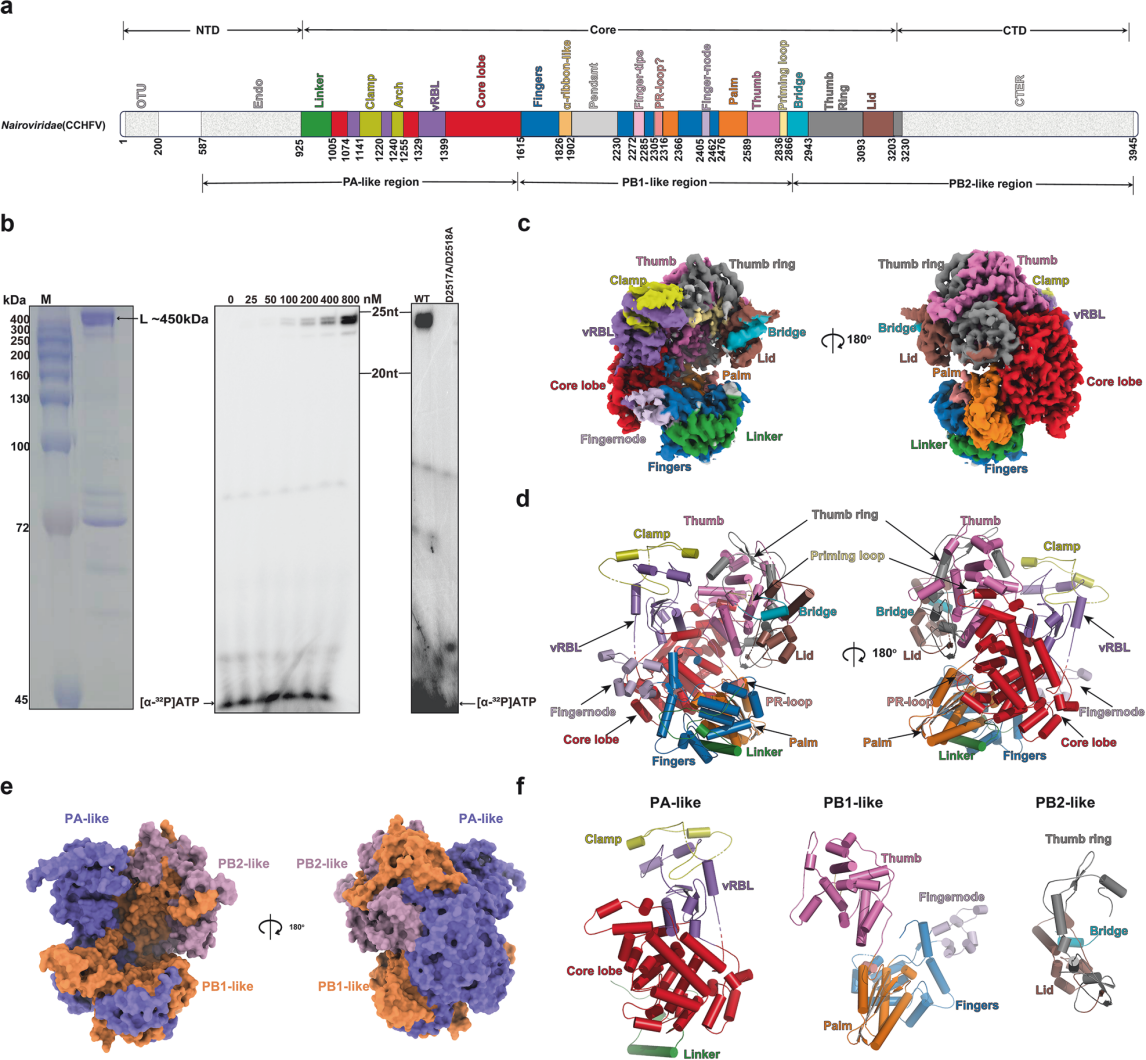
Overall architecture of the CCHFV L protein. **a** Domain organization of the CCHFV L protein. Schematic representation of the primary structure of the nairovirus CCHFV L protein, highlighting the PA-like, PB1-like, and PB2-like regions and the major structural elements resolved in the cryo-EM map. Functionally important motifs and insertions — including the core lobe, vRBL, fingers, finger node, α-ribbon, pendant domain, bridge, lid, and thumb ring — are color-coded and mapped onto the linear domain architecture. **b** Purification and in vitro activity of the full-length CCHFV L protein. Left, SDS-PAGE analysis of purified recombinant CCHFV L (~450 kDa). Middle, ^32^P-labeled radionucleotide extension assay showing concentration-dependent RNA synthesis by WT CCHFV L in the presence of the 3′ template vRNA and 5′ vRNA. Right, the catalytic mutant D2517A/D2518A does not produce detectable extension products. **c** Cryo-EM reconstruction of apo CCHFV L polymerase. Two views are presented with a 180° rotation. Major structural elements, including the core lobe, palm, fingers, finger node, vRBL, clamp, bridge, lid, thumb and thumb ring, and linker region, are illustrated. **d** Ribbon representation of the apo CCHFV L structure. Key structural elements are labeled consistent with those in **c**. Dashed outlines highlight regions that exhibit partial disorder in the apo state. **e** Modular organization of the CCHFV L polymerase. Surface representation highlighting the tripartite arrangement of the PA-like (blue), PB1-like (orange), and PB2-like (purple) regions. Two opposing orientations are shown to illustrate the overall packing of the three lobes. **f** Ribbon representation of the PA-like, PB1-like, and PB2-like modules. The core lobe, vRBL, clamp, and linker in the PA-like region; the catalytic palm, fingers and thumb in the PB1-like region; and the bridge, lid, and thumb ring in the PB2-like region are shown.

We successfully established an optimized expression system to produce the full-length CCHFV L protein (~450 kDa) in human embryonic kidney 293F (HEK293F) cells (Fig. 1a). The protein was purified to homogeneity by affinity chromatography (Fig. 1b). To confirm that the purified full-length CCHFV L protein is enzymatically active and that RNA synthesis is stimulated by the 5′ RNA as described previously in the other bunyavirus RdRp, we performed a ^32^P-labeled radionucleotide extension assay using both the 3′ template vRNA and the 5′ vRNA, as previously described^27,36,37^. Under these conditions, the purified wild-type (WT) CCHFV L protein produced a prominent extension product corresponding to the expected full-length RNA product, and the abundance of this product increased in a protein concentration-dependent manner (Fig. 1b). In contrast, the D2517A/D2518A active-site mutant did not support RNA synthesis, confirming that the observed activity depends on an intact polymerase catalytic center (Fig. 1b). The RNA products observed in this primer-independent genome replication assay were slightly larger than expected, which is consistent with previous observations for other bunyavirus polymerases in vitro^27,36,37^. These results demonstrate that the purified recombinant full-length CCHFV L protein is catalytically active in vitro and that the 5′ RNA promotes the activation of the polymerase for RNA synthesis. We therefore proceeded to prepare this sample for single-particle cryo-electron microscopy (cryo-EM) data collection. We ultimately reconstructed the apo structure of the CCHFV L protein at a global resolution of 2.85 Å (Supplementary Fig. S1 and Table S1), revealing the overall organization of the polymerase core, which is globally conserved among sNSV polymerases determined thus far^21,22,26,33,34^. The final model spans residues 925–3230, encompassing the endonuclease linker region, the core lobe, the vRNA-binding lobe (vRBL), and the RdRp core, including the canonical fingers, palm, and thumb subdomains, as well as the bridge, thumb ring, and lid domains (Fig. 1a). However, the N-terminal OTU protease and endonuclease domains, as well as the C-terminal distal region, were not observed in the cryo-EM density, likely because of their intrinsic conformational flexibility. Additionally, the large pendant insertion region (residues 1902–2230) was visible only at low map thresholds, indicating substantial local disorder.

The CCHFV L protein displays a tripartite modular organization reminiscent of that of other *Bunyavirales* and influenza virus polymerases^16^ (Supplementary Figs. S2–S4). It consists of an N-terminal PA-like region (residues 587–1615), a central PB1-like region (residues 1616–2866; corresponding to the RdRp core), and a C-terminal PB2-like region (residues 2867–3945) (Fig. 1a, c–f). Structure-based sequence alignment with representative *Bunyavirales* polymerases revealed that the CCHFV L protein core adopts a compact, globular core architecture encompassing the entire PB1-like region, the C-terminal portion of the PA-like region, and the N-terminal segment of the PB2-like region (Supplementary Fig. S2). Despite pronounced differences in primary sequence and molecular mass, the overall fold of the CCHFV L core shows striking structural conservation with that of polymerases from other sNSVs (Supplementary Figs. S2–S4).

The PA-like region (residues 925–1615) forms the N-terminal portion of the resolved CCHFV L structure and adopts a bilobed architecture characteristic of *Bunyavirales* polymerases (Fig. 1a, f). This region comprises a predominantly α-helical core lobe, which is notably larger than in any previously reported bunyavirus L protein and provides structural reinforcement to the palm and thumb domains of the RdRp core. Adjacent to it lies the vRBL, corresponding to the RNA-binding modules found in *Peribunyaviridae*, *Hantaviridae*, and *Phenuiviridae* L proteins and analogous to the “pyramid” and “base” subdomains described in *Arenaviridae* polymerases. The vRBL contains central β-sheets flanked by α-helices on both sides, forming a conserved scaffold that mediates viral RNA recognition. On one side of this region, a helical element known as the clamp is predicted to engage the 3′ terminus of the viral RNA (Fig. 1a, f), whereas on the opposite face, a long loop corresponding to the PA-arch, which in influenza polymerase interacts with the 5′ RNA end, appears to be partially disordered and unresolved in the present map.

The PB1-like region (residues 1616–2866) constitutes the catalytic RdRp core of the CCHFV L protein and adopts the canonical right-hand architecture characteristic of sNSV polymerases (Fig. 1a, f). This region comprises three principal subdomains — the fingers, palm, and thumb — which together form the polymerase active-site cavity. Both the fingers and palm subdomains exhibit bipartite architectures, each composed of two sequence-discontinuous yet structurally integrated segments, a hallmark of sNSV polymerases (Fig. 1a). Within the finger subdomain, a prominent finger node (residues 2405–2462) extends from the core and is implicated in binding the 5′ vRNA hook, which is consistent with analogous structural elements observed in other *Bunyavirales* polymerases. An additional α-ribbon insertion (residues 1826–1902), projecting from the finger domain as two long and two short α-helices, appears flexible and is unresolved in the present density map, likely reflecting intrinsic conformational mobility. The palm subdomain contains highly conserved polymerase motifs A–F, which form the catalytic center responsible for coordinating the RNA template and incoming NTPs. The prime-and-realign loop, located within the palm, is clearly resolved and contributes to shaping the catalytic cavity (Fig. 1d), whereas the priming loop, which is essential for de novo RNA synthesis initiation, remains disordered in the apo state but is expected to adopt an ordered conformation upon promoter binding, which is consistent with the conformational transitions observed in other *Bunyavirales* polymerases^16^. The thumb subdomain, positioned opposite the fingers and palm, provides a rigid structural framework that stabilizes the polymerase during RNA chain elongation (Fig. 1f). Overall, the CCHFV PB1-like region contains a globally conserved catalytic core among *Bunyavirales* polymerases but retains several unresolved, flexible structural elements in the absence of promoter RNA, suggesting that the captured conformation represents an inactive, preinitiation state of the polymerase.

The PB2-like region (residues 2867–3945) constitutes the C-terminal module of the resolved CCHFV L core and comprises the bridge, thumb ring, and lid subdomains (Fig. 1a, c–f). Notably, ~60% of the residues within this region are unresolved, likely reflecting substantial intrinsic flexibility, a characteristic also observed in the C-terminal domains of other *Bunyavirales* polymerases^16^. The bridge (residues 2866–2943) connects the thumb and thumb-ring elements, and the absence of well-defined density for most of this region suggests a high degree of intrinsic flexibility (Fig. 1c, f). The thumb ring (residues 2944–3093) encircles the thumb subdomain at the C-terminal PB2-like region, linking it to the bridge and lid domains. It serves as a structural scaffold that stabilizes the polymerase core. Situated above the product exit channel, the lid subdomain (residues 3094–3203) appears to be positioned to facilitate strand separation between the template and product RNAs, thereby promoting efficient release of the newly synthesized RNA, akin to that proposed for influenza virus polymerase^38^.

A large pendant insertion domain (residues 1902–2230) extends from the PB1-like core and is unique to the *Nairoviridae* family. Although weak density corresponding to this region was detected at lower map thresholds, its pronounced conformational flexibility precluded atomic modeling. This domain lacks a structural counterpart in any other *Bunyavirales* polymerase and may represent a family-specific regulatory element involved in modulating nairovirus replication or transcription. The structural and functional implications of this unique insertion are discussed in the following section.

Structural comparisons indicate that the overall tripartite organization of sNSV polymerases is conserved, with CCHFV L adopting the same broad PA-like, PB1-like, and PB2-like architecture observed in other bunyavirus L proteins and in influenza polymerase (Supplementary Fig. S4). In all of these enzymes, the PB1-like region forms the catalytic RdRp core, whereas the PA-like and PB2-like regions surround and regulate the polymerase chamber. This shared architectural principle has been recognized as a unifying feature of bunyavirus and influenza polymerases despite their low overall sequence similarity. However, CCHFV L also displays several nairovirus-specific elaborations. In the PA-like region, the resolved core contains an expanded core lobe and vRBL together with an unusually long endonuclease linker module, which is consistent with the exceptional size and domain complexity that distinguish nairovirus polymerases from other bunyaviral families (Supplementary Fig. S4). The PB1-like region retains the canonical catalytic right-hand fold and the conserved RdRp framework shared across bunyaviruses but is further elaborated by additional insertions, most prominently the enlarged pendant domain and associated flexible elements, which are not observed in other bunyavirus polymerases. In contrast, the PB2-like region remains the most structurally divergent and dynamic part of the enzyme, in agreement with comparative analyses across *Bunyavirales* showing that the C-terminal region is a major site of lineage-specific diversification and conformational plasticity. In CCHFV, this region is only partially resolved, which is consistent with substantial intrinsic mobility. Comparison with influenza polymerase further highlights that the principal evolutionary differences are concentrated not in the catalytic core but in the peripheral and family-specific domains, particularly in the C-terminal region beyond the mid-link and cap-related elements. In influenza virus, these include the 627 and NLS domains of PB2. Across bunyaviruses, these C-terminal modules are highly variable and may include a 627-like domain, zinc-binding domain, lariat domain, or other family-specific appendages that stabilize distinct conformational states and communicate with the polymerase core^16^. In addition, influenza and bunyavirus polymerases differ in terms of promoter organization and initiation strategy: bunyaviruses generally use internally initiated prime-and-realign replication, whereas influenza polymerase combines terminal and internal initiation depending on the template^16^. In CCHFV, the unresolved C-terminal region likely belongs to this same class of highly mobile regulatory modules, although a bona fide cap-binding domain has not yet been defined for the nairovirus polymerase.

Taken together, these observations suggest that CCHFV L combines a conserved sNSV catalytic scaffold with an unusually expanded nairovirus-specific peripheral architecture. This organization indicates that evolutionary diversification of these polymerases occurred primarily through accretion and remodeling of accessory domains rather than through substantial alteration of the core RdRp fold.

### 5′ hook vRNA bound CCHFV L

**Fig. 2:**
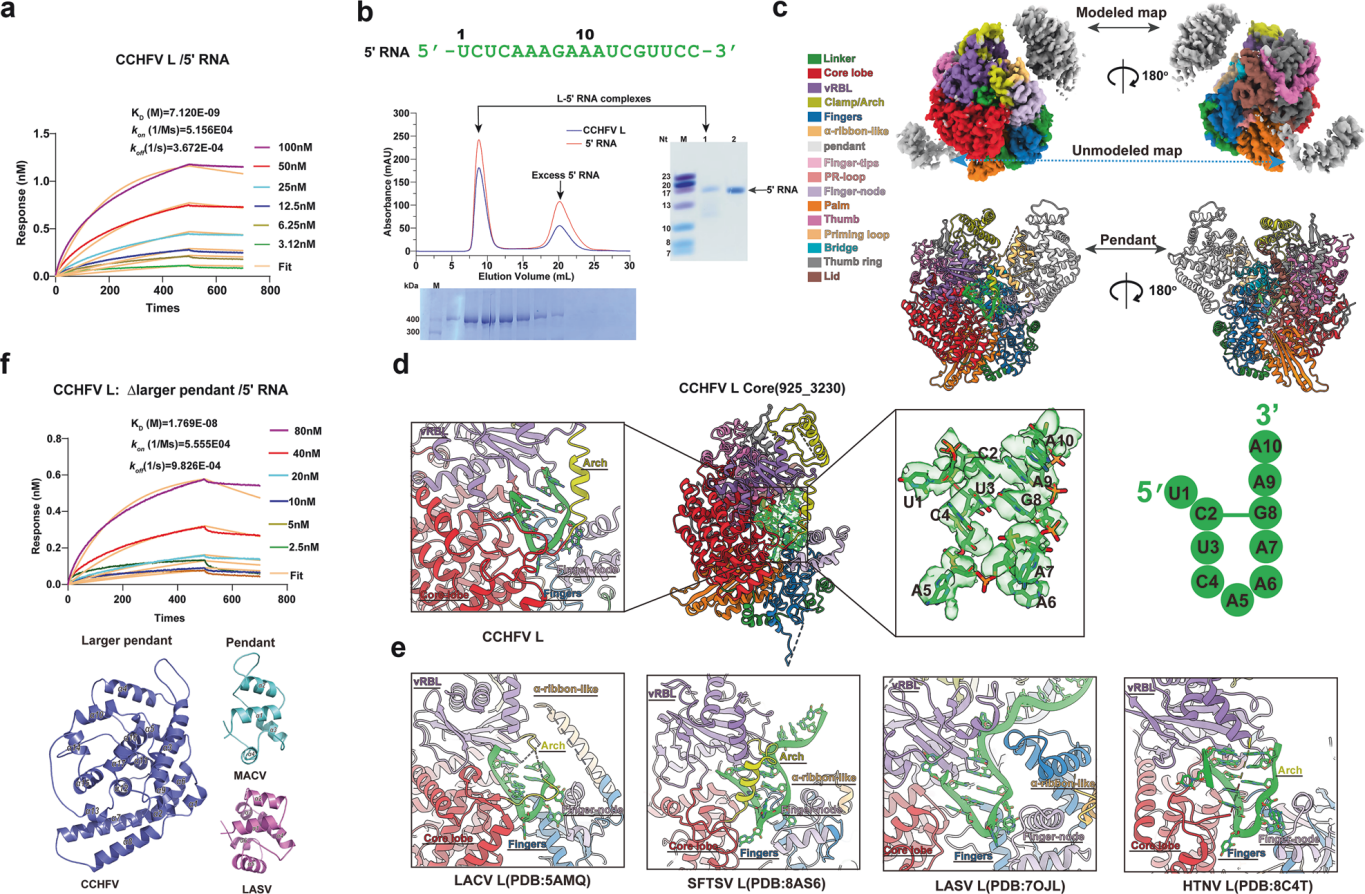
Structural basis of 5′ vRNA recognition by CCHFV L polymerase. **a** Biolayer interferometry (BLI) analysis of CCHFV L binding to 5′ vRNA. Representative sensorgrams showing the concentration-dependent association of purified CCHFV L with 18-nt 5′ viral RNA. The fitted curves yield an equilibrium dissociation constant of 7.12 nM, demonstrating high-affinity 5′ RNA recognition. **b** Assembly and purification of the CCHFV L–5′ vRNA complex. Size-exclusion chromatography profile of full-length CCHFV L incubated with a 3-fold excess of 5′ vRNA, showing the formation of a stable L–RNA complex. SDS-PAGE and urea-PAGE analyses of the corresponding fractions confirmed the comigration of L protein and RNA. The 5′ RNA sequence is shown at the top, with nucleotides 1–10 adopting the hook conformation in the final structure. **c** Cryo-EM reconstruction of the CCHFV L–5′ vRNA complex. The upper panels display the cryo-EM density maps, whereas the lower panels show the corresponding ribbon representations fitted into the density map. The pendant domain is modeled on the basis of visible density, whereas the C-terminal region is not modeled because of insufficient map support. **d** Atomic model of the 5′ vRNA hook bound within the CCHFV L core. Left: the RNA is positioned within a positively charged cleft framed by the core lobe, vRBL, fingers, arch and finger node. Middle and right: cryo-EM density and model of the first ten nucleotides (U1–A10), revealing the conserved hook architecture stabilized by one canonical base pair (C2–G8). A cartoon schematic of the hook is shown on the right. **e** Comparison of 5′ hook binding across representative sNSV polymerases. Structural comparison of the CCHFV 5′ vRNA hook with the corresponding regions in the LACV, SFTSV, LASV, and HTNV. Conserved structural elements, including the vRBL, fingers, clamp/arch, and finger node, form a shared RNA-binding pocket across polymerase families, although the surrounding architecture varies among viral lineages. **f** Functional contribution of the pendant domain to promoter binding. Top: BLI analysis of a pendant-domain deletion mutant shows a modest (~2.5-fold) reduction in binding affinity relative to that of WT L, indicating that the pendant region is not essential for 5′ vRNA engagement. Bottom: structural comparison of the enlarged pendant domain in CCHFV L with the corresponding insertions in the MACV and LASV polymerases. CCHFV has a uniquely expanded, multihelical pendant region, which is consistent with its proposed regulatory role during template loading or exit.

The 3′ and 5′ termini of bunyavirus genomes are highly conserved within viral families and exhibit strong complementarity, forming the basis for promoter recognition by viral RdRp^16^. Previous structural studies of sNSVs have identified distinct 3′ and 5′ RNA-binding sites in polymerases from LACV (*Peribunyaviridae*)^21–23^, MACV (*Arenaviridae*)^34^, LASV (*Arenaviridae*)^34,35^, SFTSV (*Phenuiviridae*)^25^, HTNV (*Hantaviridae*)^30,32^, SNV (*Hantaviridae*)^29^, TSWV (*Tospoviridae*)^28^, and the influenza virus polymerase complex^39^. The 5′ terminal nucleotides of bunyavirus genomes adopt a compact stem-loop configuration, referred to as the 5′ hook, which is essential for polymerase activation^16^. To investigate the mechanism of 5′ promoter recognition in CCHFV, we performed biolayer interferometry (BLI) using biotin-labeled RNA fragments corresponding to the terminal 18 nucleotides of the 5′ viral RNA (5′ vRNA₁–₁₈). Under these conditions, the purified CCHFV L protein exhibited robust binding to 5′ vRNA₁–₁₈, with an equilibrium dissociation constant (*K*_d_) of 7.12 nM (Fig. 2a). This affinity is comparable to that reported for the LACV L protein binding to the 5′ genomic extremities (*K*_d_ = 13.8 nM)^21^, confirming the presence of a high-affinity 5′ RNA-binding site in CCHFV L. Subsequently, purified CCHFV L protein was incubated with a 3-fold molar excess of an 18-mer synthetic RNA corresponding to the 5′ terminus (5′-UCUCAAAGAAAUCGUUCC-3′) on ice for 30 min, resulting in efficient complex formation, as verified by SDS-PAGE and urea-PAGE (Fig. 2b). The assembled L–RNA complex was immediately used for cryo-EM grid preparation. The cryo-EM structure of the CCHFV L–5′ vRNA complex was resolved at 3.76 Å (Supplementary Fig. S5 and Table S1). Owing to the limited resolution of the map, we were only able to trace the backbone of the 5′ RNA, while the orientations of individual bases could not be confidently assigned (Fig. 2c). In addition, we successfully fitted the pendant domain model of the CCHFV L protein to the cryo-EM map (Fig. 2c). However, the C-terminal region (C-domain) exhibited weak density at low map thresholds, making it difficult to obtain a reliable atomic model for this region. Furthermore, the N-terminal segments of the polymerase remained unresolved and displayed a disordered state similar to that observed in the apo form of the polymerase, indicating significant flexibility in these regions.

To obtain a more accurate and detailed visualization of the 5′ RNA trajectory and base orientation, we optimized multiple RNA constructs of varying lengths for complex formation with the CCHFV L protein and subsequent cryo-EM analysis. Despite extensive efforts, side-chain features of the RNA bases remained unresolved in the full-length reconstructions. In both the apo and 5′ RNA-bound full-length L structures, only the polymerase core displayed a well-defined density, whereas the N- and C-terminal regions were largely disordered, likely due to pronounced conformational heterogeneity. This observation indicated that the core region constitutes the most rigid and structurally stable module of the polymerase. On the basis of this insight, we designed a truncated construct comprising only the core region (residues 926–3230). We confirmed that this core construct retained in vitro RNA synthesis activity, although at a lower level than the WT protein did (Supplementary Fig. S6), and subsequently reconstituted it with the 5′ RNA for single-particle cryo-EM analysis. The resulting dataset exhibited improved particle homogeneity and angular distribution, enabling reconstruction of the complex at 3.32 Å resolution (Fig. 2d; Supplementary Fig. S7 and Table S1). The refined map revealed a continuous and well-defined density for the phosphate backbone, ribose, and base moieties of the 5′ RNA, allowing unambiguous modeling of the entire hook-like conformation within a positively charged cleft framed by the vRBL, core lobe, finger domain, and finger node (Fig. 2d). On the basis of the cryo-EM density, the first ten nucleotides of the CCHFV 5′ RNA adopted a characteristic hook-like conformation (Fig. 2d), whereas nucleotides 11–18 appeared flexible and were not visible in the map. The 5′ RNA hook is stabilized by one canonical base-pairing interaction between C2 and G8. This stabilization pattern closely resembles that of LASV^35^ and SFTSV^25^ hook polymerases, which also feature one canonical base pair supporting the hook architecture (Supplementary Fig. S8a), but differs from the HTNV^31^ and LACV^22^ hook polymerases, in which two or three canonical base pairs reinforce the hook conformation (Supplementary Fig. S8b). In addition to the C2–G8 interaction, several amino acid side chains from the fingers, finger node, and core lobe further contribute to the stabilization and coordination of the 5′ RNA hook within the binding cleft. The RNA-binding site is capped by an arch-like structural element that extends from the vRBL, fully enclosing the 5′ RNA and likely securing it in place during promoter recognition and polymerase activation.

To further examine the contribution of the 5′ RNA hook to binding, we performed binding experiments using a panel of mutant 5′ RNA constructs targeting the first four nucleotides, since these nucleotides were initially thought to contribute most prominently to both the protein–RNA binding interface and the intramolecular interactions that stabilize the 5′ RNA hook conformation (Supplementary Fig. S9). Importantly, the map quality was sufficiently clear to enable reliable analysis of protein‒RNA interactions. BLI analysis revealed that most single-nucleotide substitutions within the first four nucleotides have little or only a modest effect on binding affinity (Supplementary Fig. S10). In particular, the absence of a pronounced binding defect upon C2 mutation indicated that the C2–G8 base pair is not essential for binding but instead plays a supportive structural role. These findings suggest that binding is not primarily dictated by strict base-specific recognition at these positions but rather by nonsequence-specific interactions with the RNA phosphate backbone and recognition of the local RNA architecture and shape, as is commonly observed in many protein‒RNA interfaces. In such contexts, single-nucleotide substitutions are generally well tolerated unless they substantially perturb the local RNA structure. Notably, several mutations, particularly U3C, U3G, and substitutions at position C4 (e.g., C4A), markedly enhanced binding affinity to the picomolar range. From a structural perspective, these mutations may stabilize the local 5′ RNA conformation, improve base stacking, or reduce the conformational flexibility of the hook-like structural element, thereby promoting a more preorganized RNA geometry for binding (Supplementary Fig. S9). In addition, substitution of U3 with C or G may alter the local hydrogen-bonding potential and electrostatic environment, which may in turn reinforce the intramolecular hydrogen-bonding network of the RNA and increase local structural stability. Similarly, mutations at position C4 may reshape local stacking interactions and nucleotide positioning, thereby generating a more favorable RNA surface for recognition by R1551 (Supplementary Fig. S9).

Taken together, these results suggest that the contribution of the 5′ RNA hook to binding is primarily determined by its overall local architecture rather than strict base identity. The C2–G8 base pair likely supports the structural integrity of this region, whereas U3 and C4 modulate binding by influencing local conformation, stacking interactions, and interface geometry. Thus, the functional role of this region is best understood in structural rather than sequence-specific terms. More broadly, this binding mode closely resembles the promoter recognition observed in other sNSV polymerases (Fig. 2e), supporting a conserved mechanism of 5′ RNA-mediated polymerase regulation across sNSVs^16^.

Notably, in the 5′ hook-bound structure of the CCHFV L protein, we identified a unique and enlarged pendant insertion domain located between the finger domain and the α-ribbon helices (Fig. 2f). This feature, which corresponds topologically to the arenavirus-specific insertion domain, is absent in other sNSV polymerases but is highly conserved among *Nairoviridae* members (Supplementary Figs. S2, S11). The larger pendant domain comprises ~15 α-helices, making it substantially larger than the corresponding domain in the MACV and LASV polymerases, which contain only 4–5 α-helices. This region was not resolved in the apo structure of CCHFV L, indicating considerable conformational flexibility. To assess whether the larger pendant domain contributes to promoter recognition, we performed BLI analysis using a deletion mutant lacking this domain. Compared with the WT L protein, the mutant exhibited a modest, ~2.5-fold reduction in binding affinity, with a dissociation constant *K*_d_ of 17.6 nM (Fig. 2f). These results suggest that the larger pendant domain has a minor effect on the affinity of the L protein for 5′ vRNA and is likely not essential for promoter engagement (Fig. 2f). This finding is consistent with findings from MACV and LASV polymerases, in which deletion of the corresponding pendant domain did not alter binding affinity for either 3′ vRNA or 3′ cRNA^34^, further supporting that this region is not directly involved in promoter recognition. Together, these findings indicate that the larger pendant domain is dispensable for promoter recognition, suggesting that it may instead play a role in subsequent stages of the polymerase catalytic cycle or in interactions with other viral or host components.

### Conformational rearrangements and activation upon 5′ vRNA binding

**Fig. 3:**
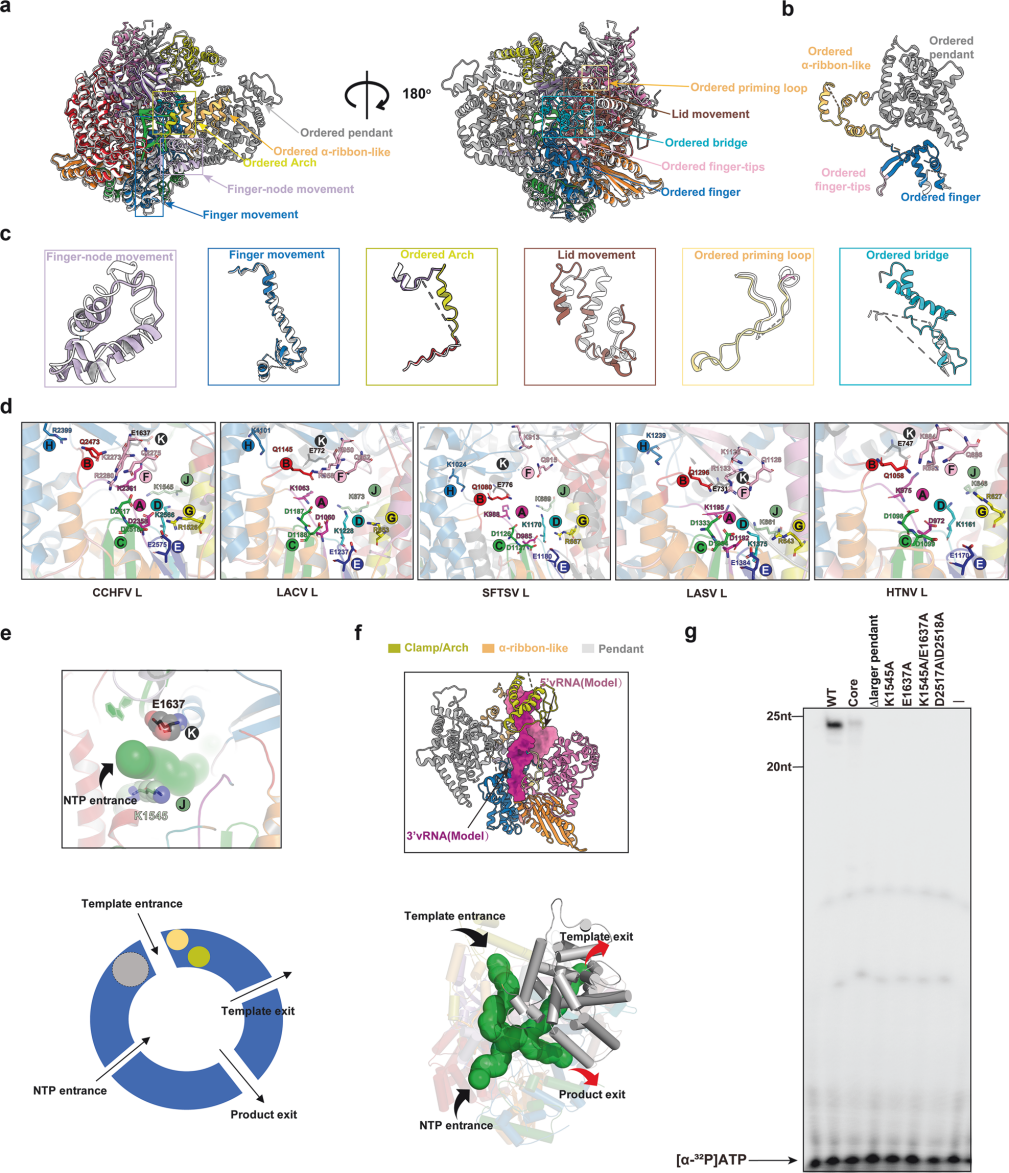
Conformational rearrangements and activation of CCHFV L polymerase upon 5′ vRNA binding. **a**–**c** Structural comparison between apo and 5′ vRNA-bound CCHFV L, showing global conformational rearrangements upon promoter binding. Superposition of the apo and 5′ vRNA-bound models revealed significant compaction of the polymerase, accompanied by coordinated movements of the fingers, finger node, and lid domains toward the active site. Additionally, disorder-to-order transitions are observed in several regions, including the priming loop, bridge, arch, pendant domain, fingertips, and α-ribbon-like element. These conformational changes are critical for the activation of the polymerase during RNA synthesis. **d** Functional motifs (A–H) within the catalytic center of CCHFV L and comparison with those of other *Bunyavirales* polymerases. The identified motifs J and K, conserved across all *Bunyavirales*, define a constriction at the NTP entry channel. **e** Details of the NTP entry channel, showing that residues K1545 and E1637 form a constriction, which may regulate NTP selection during RNA synthesis. **f** Proposed regulatory role of the pendant domain in template entry and exit. Structural mapping suggests that the pendant domain is positioned near the template entry and/or template exit channel, potentially modulating RNA synthesis by regulating template loading and release. **g** In vitro RNA synthesis of WT and mutant CCHFV L proteins, including WT, core, Δpendant, K1545A, E1637A, K1545A/E1637A, the catalytic-dead mutant D2517A/D2518A, and the no-protein control (–).

To define how promoter engagement activates CCHFV L, we compared the apo and 5′ vRNA-bound L structures (Fig. 3a–c). Global superposition of the two models revealed that 5′ vRNA binding induces marked compaction of the polymerase, with coordinated movements of the fingers, finger-node and lid domains toward the active site (Fig. 3a–c). Promoter binding also occurs in several regions that are largely invisible in the apo map. In the 5′ vRNA-bound complex, robust density appears for the α-ribbon-like insertion and the pendant domain, as well as for the distal parts of the fingers and the bridge (Fig. 3b–c). Local overlays highlight a hinge-like motion of the finger-node and fingers, which swing inward to cradle the 5′ hook RNA (Fig. 3c). Moreover, the arch segment emerging from the vRBL folds into an extended helix-turn-helix element that closes over the RNA cleft, acting as a clamp that locks the promoter in place (Fig. 3c). On the C-terminal side of L, the lid undergoes a marked rearrangement, rotating toward the exit channel, while the bridge becomes fully ordered (Fig. 3c). Concomitantly, two catalytic regulatory elements — the fingertips (motif F) and the priming loop — undergo a transition from being largely disordered in the apo enzyme to adopting well-defined conformations in the 5′ vRNA-bound state (Fig. 3b, c), in agreement with observations previously reported for LACV^21^, influenza A^39^ and TSWV^28^ L. In this active conformation, motif F, the priming loop and the newly ordered bridge pack tightly against the conserved motifs A–E, G and H, completing the assembly of a closed, catalytically competent active site. Together, these concerted domain motions and disorder-to-order transitions explain how 5′ promoter binding remodels CCHFV L from a relaxed apo conformation to a preinitiation state poised for RNA synthesis.

Like other sNSV polymerases, the CCHFV L protein contains a canonical catalytic center composed of eight conserved motifs (A–H), a hallmark of *Bunyavirales* RdRps. Structural comparison revealed that the architecture and spatial arrangement of these motifs are highly conserved across representative *Bunyavirales* polymerases, including those from LACV, SFTSV, LASV, and HTNV (Fig. 3d), underscoring a shared evolutionary and mechanistic framework for RNA synthesis within this viral order. During our structural analyses, we identified two previously unrecognized residues, K1545 and E1637, situated within the core lobe and finger domains, respectively. These residues form a constriction that defines the gateway of the NTP entry channel (Fig. 3e), a region that has not been functionally characterized in *Bunyavirales* polymerases. Sequence and structural alignments revealed that both positions are strictly conserved across all available *Bunyavirales* L proteins (Fig. 3d; Supplementary Fig. S2). These observations prompted us to define this region as motifs J and K (Fig. 3d, e) and to propose that they function as regulatory elements governing NTP selection and entry into the RdRp active site.

To evaluate the functional importance of the J and K motifs in the proposed NTP entry pathway, we introduced alanine substitutions at two residues lining the channel, K1545 and E1637, individually and in combination and examined their activities using an in vitro RNA synthesis assay. Under the same protein concentration and exposure conditions as those used for the WT enzyme, the K1545A, E1637A, and K1545A/E1637A mutants showed a dramatic reduction in RNA synthesis activity, and the RNA products were barely detectable (Fig. 3g). Their signals were nearly indistinguishable from those of the catalytic-dead mutant D2517A/D2518A and the no-protein control, indicating that these substitutions severely compromised polymerase function under the assay conditions. These results show that K1545 and E1637 are functionally important for polymerase activity and are consistent with our structural model in which the J/K motifs contribute to the architecture of the proposed NTP entry pathway.

Collectively, our findings establish motifs J and K as previously unrecognized yet integral components of the CCHFV L polymerase. By modulating NTP access to the catalytic center, these motifs likely play a critical role in coordinating the RNA synthesis cycle. Their strict conservation highlights a fundamental mechanism underlying *Bunyavirales* replication.

To better elucidate the functional contribution of the large pendant domain in CCHFV L, we first examined whether this domain influences vRNA positioning. Previous BLI assays revealed that deletion of the pendant domain caused a moderate, ~2–3-fold reduction in 5′ RNA binding affinity relative to that of the WT L protein (Fig. 2f), suggesting that this region does not markedly alter the intrinsic affinity for 5′ RNA. To more precisely assess the spatial relationship between the pendant domain and viral RNA, we superimposed the LACV initiation-state structure containing both 3′ and 5′ RNA (PDB: 7ORN) onto the CCHFV–5′ RNA complex. The resulting model revealed that neither RNA strand directly contacts the pendant domain (Fig. 3f), indicating that this region is not part of the canonical RNA-binding interface. Internal tunnel analysis and structural mapping further revealed that the pendant domain is positioned adjacent to the putative template-entry and/or template-exit channel — a critical conduit through which genomic RNA is threaded into and released from the RdRp active site (Fig. 3f). This spatial proximity suggests that the pendant domain may regulate template loading or release during the catalytic cycle.

To assess the functional importance of the larger pendant domain, we analyzed a truncation mutant (Δlarger pendant) using an in vitro RNA synthesis assay. While the activity of the WT enzyme was robust, compared with the catalytic-dead mutant and the no-protein control, the truncation mutant exhibited a dramatic loss of RNA synthesis activity (Fig. 3g). These data demonstrate that the pendant domain is essential for polymerase function. Given its proximity to the proposed template entry/exit channel but lack direct RNA contact, the pendant domain likely contributes to regulating template handling during RNA synthesis. Together, these data demonstrate that although the large pendant domain does not directly contact viral RNA, it likely functions as a crucial regulator of CCHFV polymerase activity, possibly by modulating template entry or exit within the internal tunnel to ensure accurate initiation and productive RNA synthesis.

## Discussion

**Fig. 4:**
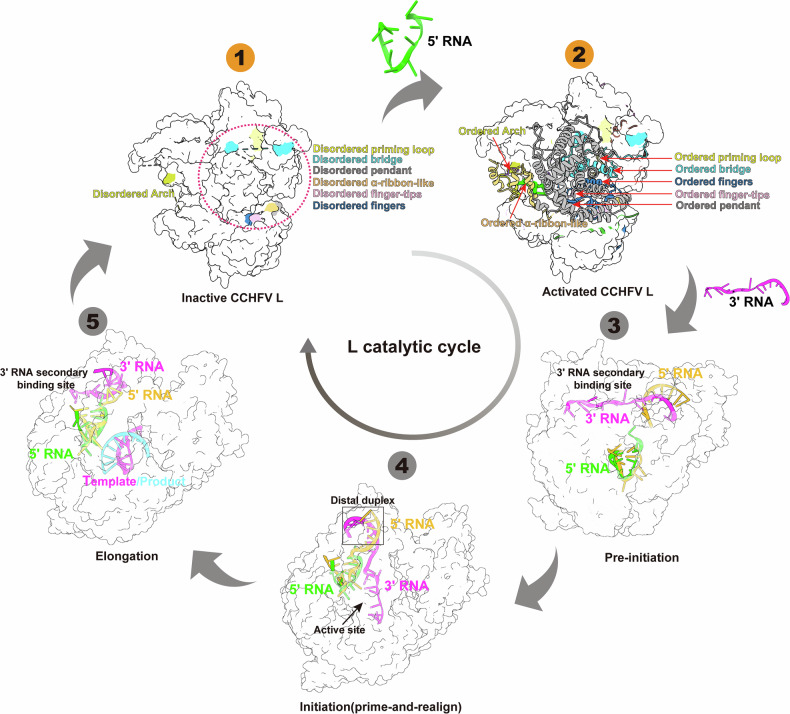
Proposed catalytic cycle of CCHFV L polymerase, with LACV L as the primary structural reference. In the absence of RNA, CCHFV L adopts an inactive and highly flexible conformation with multiple disordered functional elements. Binding of the 5′ vRNA stabilizes a hook conformation and induces the ordering of key structural elements, thereby promoting polymerase activation. Subsequent recruitment of the 3′ template, distal duplex formation, and positioning within the RdRp active site enable initiation, likely via a prime-and-realign mechanism. Transition to elongation involves disruption of the distal duplex, formation of an ~8–10 bp template–product duplex, and coordinated domain rearrangements. The 3′ template may be transiently captured by a secondary binding site, facilitating template handling and polymerase recycling.

In this study, we report the structural characterization of the largest known L protein among *Bunyavirales*, providing the first structural framework for understanding polymerase organization and regulation in nairoviruses. Recent structural studies support a broadly conserved model in which bunyavirus L progresses through promoter recognition, preinitiation, initiation, and elongation, although family-specific differences remain^16,21–23,25,30,32,34,35^ (Fig. 4). In many bunyavirus polymerases, productive promoter engagement involves 5′ hook binding, distal duplex formation, and recruitment of the 3′ template to the RdRp active site. In the absence of RNA, L can adopt an inactive conformation. Productive engagement begins when the conserved genome ends bind in distinct, sequence-specific sites: the 5′ vRNA binds as a hook on the polymerase surface, whereas the 3′ vRNA binds either along the active-site trajectory or in a distinct 3′ secondary binding site. Although the genome termini are highly complementary, the terminal ~10 nucleotides of the 5′ end generally form the hook, whereas the 3′ terminus is captured as a single strand; nucleotides further upstream form a distal duplex. This distal duplex is a central organizing element that stabilizes active conformations and helps guide the 3′ template toward the RdRp active site. In several bunyavirus families, replication is thought to initiate internally, often through a prime-and-realign mechanism, although family-specific variation and alternative initiation modes have also been proposed. Within this framework, 5′ RNA binding promotes or stabilizes an activation-competent polymerase state and facilitates the recruitment of the 3′ end into the catalytic cavity and license initiation. Transition into elongation is accompanied by disruption of the distal duplex, progressive formation of a template–product duplex within the active site, and opening or widening of the polymerase core to accommodate RNA synthesis. The available elongation structures indicate an approximately 8–10-bp template–product duplex, together with coordinated rearrangements of auxiliary domains and loops that stabilize the elongation-competent state. A further recurring feature is the 3′ secondary binding site, formed by the vRBL or pyramid region together with thumb or thumb-ring elements. During elongation, the 3′ template exits the catalytic cavity and can be captured by this site, which may function as a protective docking platform for template handling and may facilitate polymerase resetting or recycling. Overall, the current structural evidence supports a model in which 5′ hook formation, distal duplex assembly, 3′ template recruitment, initiation, and duplex-supported elongation together define the core replication cycle. In contrast, termination and recycling remain less well defined, and direct structural evidence for these later stages is still lacking. Importantly, our current CCHFV structures capture only the earliest and most fundamental states within this continuum (steps 1–2; Fig. 4). Specifically, the apo-CCHFV L structure reveals an inactive and highly flexible state in which multiple functional elements, including the priming loop, bridge, pendant domain, α-ribbon-like element, fingers, fingertips, and arch, are not resolved or appear disordered. Upon binding of the 5′ vRNA promoter, these elements undergo coordinated ordering, allowing the 5′ terminus to adopt a stabilized hook conformation within the hook-binding pocket and thereby promoting activation of the polymerase catalytic center (Fig. 4). This promoter-driven activation mechanism is similar to that observed in other sNSVs but is executed through the uniquely expanded and complex domain architecture of nairovirus L. These findings provide fundamental insights into the molecular basis of CCHFV genome replication and transcription. Notably, the identification of nairovirus-specific structural features — particularly the extensive pendant domain and its functional coupling to template-tunnel dynamics — reveals a previously unrecognized regulatory mechanism within sNSVs.

Although the N- and C-terminal regions of CCHFV L were not resolved in our cryo-EM maps, the available biochemical and comparative structural evidence allows for cautious functional interpretation. The extreme N-terminus harbors an OTU-like protease domain, a hallmark of nairovirus polymerases that is absent from most other bunyavirus L proteins. Moreover, the region linking the OTU domain to the conserved RdRp core contains a putative leucine zipper and a C2H2 zinc finger motif, both of which are proposed to contribute to NP binding by the N-terminal region of L^40,41^. The OTU domain has been shown to possess deubiquitinase and deISGylase activities and to antagonize host innate immune signaling^14,42–47^. However, biochemical studies of the full-length CCHFV L protein have indicated that OTU-associated DUB activity and RdRp elongation can be inhibited independently in vitro, supporting the view that the OTU domain functions primarily as an accessory immune-modulatory module rather than as a direct constituent of the polymerase catalytic machinery^48^. The downstream EN domain is more directly linked to viral transcription. Broadly across bunyaviruses, the cap-snatching endonuclease is required for cap-dependent transcription rather than replication, and the positioning of this domain substantially farther downstream in the N-terminal region is unusual in nairoviruses compared with other bunyaviruses. Its functional importance in CCHFV was first demonstrated in a virus-like particle system, in which the catalytic residue D693 was shown to be required for mRNA transcription^49^. Early biochemical studies further suggested that CCHFV and related nairovirus endonucleases were catalytically inactive in vitro despite retaining metal-binding capacity through conserved acidic residues^50^. More recent structural and biochemical work, however, has indicated that nairovirus endonucleases constitute a distinct subclass characterized by Mn²⁺-dependent activity, a preference for uridine-rich RNA substrates, and a two-metal-ion catalytic mechanism important for transcription^51^. In our full-length construct, the unresolved EN domain is connected to the ordered polymerase core by a long endonuclease linker that wraps around the RdRp, supporting the idea that the EN domain is not merely appended to the enzyme but is likely dynamically coupled to the core during transitions between transcription-related states. In contrast, the C-terminal region remains less well defined. Comparative analyses across bunyaviruses indicate that the C-terminal region is among the most divergent and mobile parts of L, often undergoing large rearrangements during activation and commonly housing a CBD (or CBD-like domain) plus variable C-terminal appendages involved in transcription-associated conformational changes^16,19,23,35,52,53^. However, despite this family-wide precedent, a true CBD has not yet been identified in CCHFV L, and our current structure likewise does not resolve this region. We therefore favor a conservative model in which unresolved C-terminal region contributes to transcription-associated regulation, potentially including cap handling and/or conformational communication with the polymerase core, while the OTU domain primarily mediates host pathway modulation and the EN domain provides the cap-snatching nuclease activity required for transcription. These assignments are consistent with the available literature and our structure but should still be regarded as provisional until the full-length N- and C-terminal regions can be captured structurally and investigated functionally in the context of the intact replication complex.

Overall, the structural framework presented here therefore establishes a foundation for the rational design of CCHFV-specific polymerase inhibitors and offers valuable guidance for antiviral strategies against one of the world’s most lethal viral pathogens.

## Materials and Methods

### Plasmid construction

The coding sequence of CCHFV L (strain YL16070, GenBank accession KY354082), engineered to contain an N-terminal His tag and a C-terminal twin Strep II tag, was codon-optimized and synthesized into a pcDNA3.1 expression vector by GenScript. A CCHFV L core mutant (residues 926–3230) was generated by PCR amplification, cloned and inserted into the same pcDNA3.1 backbone while preserving the N- and C-terminal tags. A complete list of all synthesized constructs is provided in Supplementary Table S2.

### Cell culture

HEK293F cells were cultured in a humidified incubator at 37 °C with 5% CO₂ and maintained in SMM 293 T II medium (Sino Biological).

### Protein expression and purification

To express the full-length or core CCHFV L protein, HEK293F cells (1 L culture) were transfected at a density of 3 × 10^6^ cells/mL with a mixture containing 1 mg plasmid DNA and 3 mg polyethyleneimine (PEI, Polysciences). Transfected cells were maintained in suspension at 37 °C, 5% CO₂, and 130 rpm. At 72 h post transfection, the cells were harvested by centrifugation at 5000 rpm for 15 min. The resulting cell pellet was resuspended in lysis buffer (50 mM Tris-HCl, pH 8.0, 1 M NaCl, 1 mM PMSF, and 10% glycerol), lysed by sonication, and clarified by centrifugation at 20,000 rpm for 1 h. The supernatant was filtered through a 0.45 µm membrane and loaded onto Strep-Tactin XT resin. After the samples were washed with 20 column volumes of wash buffer (50 mM Tris-HCl, pH 8.0, 1 M NaCl, and 10% glycerol), the bound protein was eluted with elution buffer (50 mM Tris-HCl, pH 8.0, 150 mM NaCl, and 50 mM D-biotin). Eluted fractions were pooled, concentrated, and further purified by size-exclusion chromatography using a Superdex 200 10/300 column (GE Healthcare) in buffer containing 20 mM HEPES, pH 7.5, 100 mM NaCl, 10% glycerol and 3 mM dithiothreitol. Purified CCHFV L protein was assessed by SDS-PAGE. Peak fractions were collected for cryo-EM sample preparation, whereas the remaining protein was concentrated and flash-frozen in liquid nitrogen for subsequent BLI assays.

### Urea-PAGE analysis

RNA generated from the different expression constructs was purified by phenol‒chloroform extraction followed by ethanol precipitation, after which the pellets were resuspended in DEPC-treated water. The RNA samples were combined with denaturing loading buffer (Thermo Fisher Scientific) and resolved on a 15% urea-polyacrylamide gel in 1× TBE for 70 min, following procedures described previously^54,55^. After electrophoresis, the gels were incubated with Stains-All (Thermo Fisher Scientific) and imaged using a Bio-Rad ChemiDoc system.

### Preparation of oligonucleotides

All the oligonucleotides used in this study were chemically synthesized and purchased from GenScript. The sequences are listed in Supplementary Table S3.

### Cryo-EM sample preparation

The CCHFV L protein used for cryo-EM analysis was prepared at a concentration of ~0.9 mg/mL. To assemble the CCHFV L–5′ vRNA complex, the purified L protein was incubated with 5′ vRNA at a 1:3 molar ratio on ice for 2 h. Cryo-EM grids were prepared using an FEI Vitrobot, with the chamber maintained at 4 °C and 100% humidity. A 3.5 μL aliquot of sample was applied to a glow-discharged 300-mesh Quantifoil R1.2/1.3 gold grid, and excess liquid was removed using filter paper (blot force 2, blot time 3 s). Grids were subsequently plunge-frozen in liquid ethane pre-cooled by liquid nitrogen and stored in liquid nitrogen for long-term preservation.

### Cryo-EM data collection

Apo-state CCHFV datasets were collected using a Titan Krios microscope equipped with a Gatan K3 direct electron detector, operating at a nominal magnification of 105,000× and a pixel size of 0.83 Å. Datasets for the 5′ RNA-bound full-length or core regions of CCHFV were collected on a Titan Krios microscope equipped with a Falcon4 detector at a nominal magnification of 165,000× and a pixel size of 0.74 Å. Data were acquired at three facilities: the Center for Biological Imaging and the Core Facilities for Protein Science at the Institute of Biophysics, Chinese Academy of Sciences, the Cryo-EM Platform at Peking University, and Shuimu BioSciences. Movies were recorded using SerialEM^56,57^ or EPU software, with a cumulative electron exposure of 60 e^−^/Å^2^. Beam-induced motion was corrected using MotionCor2^58^. During data collection, defocus values were set in the range of –1.5 μm to –2.0 μm.

### Cryo-EM data analysis

For the apo-state dataset, a total of 9040 movies were recorded. Following motion correction^58^ and CTF estimation^59^ in cryoSPARC-3.3.2^60^, ~1.3 million candidate particles were extracted. Several cycles of 2D classification were carried out, and particles with well-defined features were used to train a Topaz^61^ model for improved particle selection. Subsequent ab initio reconstruction followed by iterative rounds of heterogeneous refinement yielded a class of 55,537 particles, which were subjected to nonuniform refinement to generate the final apo-state map. The datasets for the 5′ RNA-bound core region were processed following an analogous workflow.

For the full-length CCHFV L in complex with 5′ RNA, 10,155 movies were collected, from which ~1.1 million particles were initially extracted. These particles were subjected to heterogeneous refinement using six initial 3D classes. Two classes exhibiting high-quality structural features, comprising 429,156 particles, were selected for further refinement. A second round of heterogeneous refinement narrowed the dataset to ~11,000 particles. Subsequent focused 3D classification identified a well-resolved subset of 19,012 particles, which was further refined using non-uniform refinement. All the final reconstructions were post-processed and sharpened using DeepEMhancer^62^.

3D FSC analysis was performed for the apo, 5′ RNA-bound core, and 5′ RNA-bound full-length reconstructions to assess directional resolution anisotropy and map isotropy. The resulting sphericity values and directional FSC distributions are shown in Supplementary Fig. S12.

### Model building and refinement

To generate the structural model of CCHFV L, we first utilized AlphaFold3^63^ to predict the initial structure. For the generation of the CCHFV L–5′ vRNA complex, the RNA component was constructed de novo. The initial protein coordinates were rigid-body docked into the WT cryo-EM density using UCSF Chimera^64^. Model optimization began with an initial round of PHENIX^65^ real-space refinement with morphing, followed by manual rebuilding in Coot^66^, to improve local geometry and ensure accurate placement within the density. RNA nucleotides lacking sufficient cryo-EM support were removed during this step. A final cycle of PHENIX^65^ real-space refinement was performed to produce the optimized models. Representative EM densities of various complexes are shown in Supplementary Fig. S13.

### BLI assays

BLI measurements assessing the interaction between CCHFV L and 5′ vRNA were conducted on a ForteBio Octet RED96e instrument using 96-well microplates (Molecular Devices, 5085651) at 25 °C with agitation at 1000 rpm. Streptavidin biosensors (Sartorius, 18-5019) were hydrated for at least 10 min prior to use and subsequently loaded with 100 nM biotin-labeled 5′ vRNA for 300 s. After establishing a 60 s baseline in kinetic buffer (PBS), the sensors were transferred into wells containing serial 2-fold dilutions of CCHFV L for 500 s to monitor association, followed by 200 s of dissociation in kinetic buffer. Raw sensorgrams were reference-subtracted and fitted using a 1:1 binding model in ForteBio Data Analysis Software v11.1.

### In vitro RNA polymerase assays

Recombinant CCHFV L polymerase activity was assessed in a radiolabeled, primer-independent in vitro RNA synthesis assay. WT L protein was analyzed at final concentrations of 0–800 nM, whereas mutant proteins were tested at 800 nM, unless otherwise stated. Reactions contained 20 mM HEPES, pH 7.5, 100 mM NaCl, 1 mM DTT, 10% glycerol, 5 mM MgCl₂, 5 mM MnCl₂, 250 nM each of 3′ and 5′ vRNA, 1 U/μL RNasin Plus, 125 μM each of GTP, CTP, and UTP, 250 nM ATP, and 0.2 μL [α-³²P]-ATP in a total volume of  10 μL. After incubation at 30 °C for 1 h, the reactions were stopped with an equal volume of formamide loading dye, heated at 80 °C for 5 min, and analyzed on 20% denaturing polyacrylamide/6 M urea gels. Radiolabeled RNA products were detected by phosphor imaging using a Typhoon FLA 9500 scanner.

## Supplementary information

Supplementary Information

Supplementary Materials

CONSORT 2025 editable checklist

Supplementary Materials

## Data Availability

Atomic models of the CCHFV L apo, CCHFV L–5′ vRNA complex and Core–5′ vRNA have been deposited into the Protein Data Bank with the accession codes 9XSC, 9XSB, and 9XRX, respectively. The corresponding 3D cryo-EM density maps have been deposited into the Electron Microscopy Data Bank under the accession codes EMD-67173, EMD-67169, and EMD-67157.
