# Supplementary material for: Structures of the Crimean-Congo hemorrhagic fever virus RNA-dependent RNA polymerase
Source: Cell Discov. 2026 Jul 14;12:51. doi: 10.1038/s41421-026-00905-5 (PMC13365374; doi:10.1038/s41421-026-00905-5)
Supplement: Supplementary file 1 — Supplementary Information [file 41421_2026_905_MOESM1_ESM.pdf]

Supplementary Materials for

Cryo-EM structures of the Crimean-Congo Haemorrhagic Fever Virus RNA Dependent RNA Polymerase

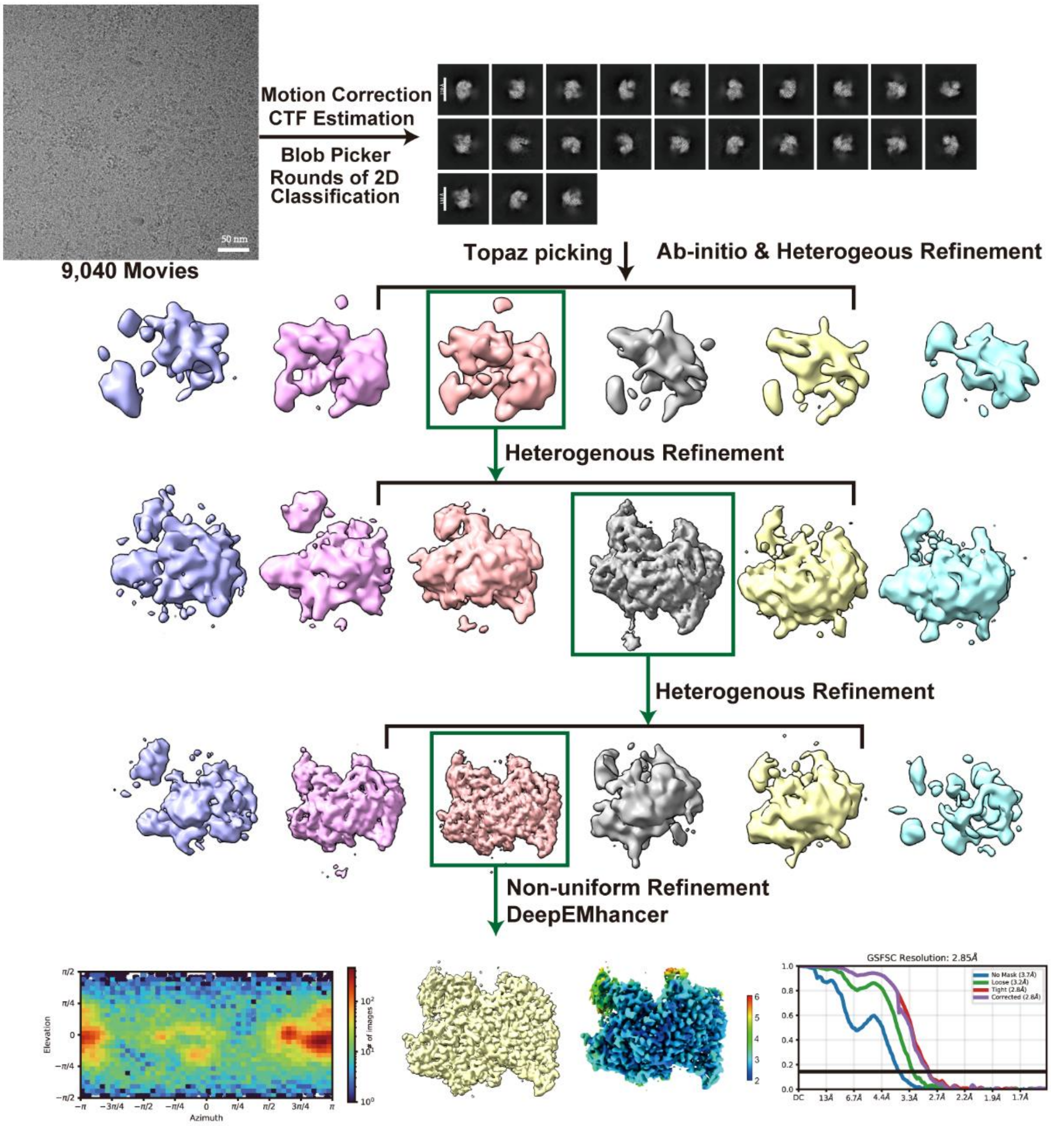

Figure S1 Data processing workflow of cryo-EM data analysis of apo structure of the CCHFV L Core.

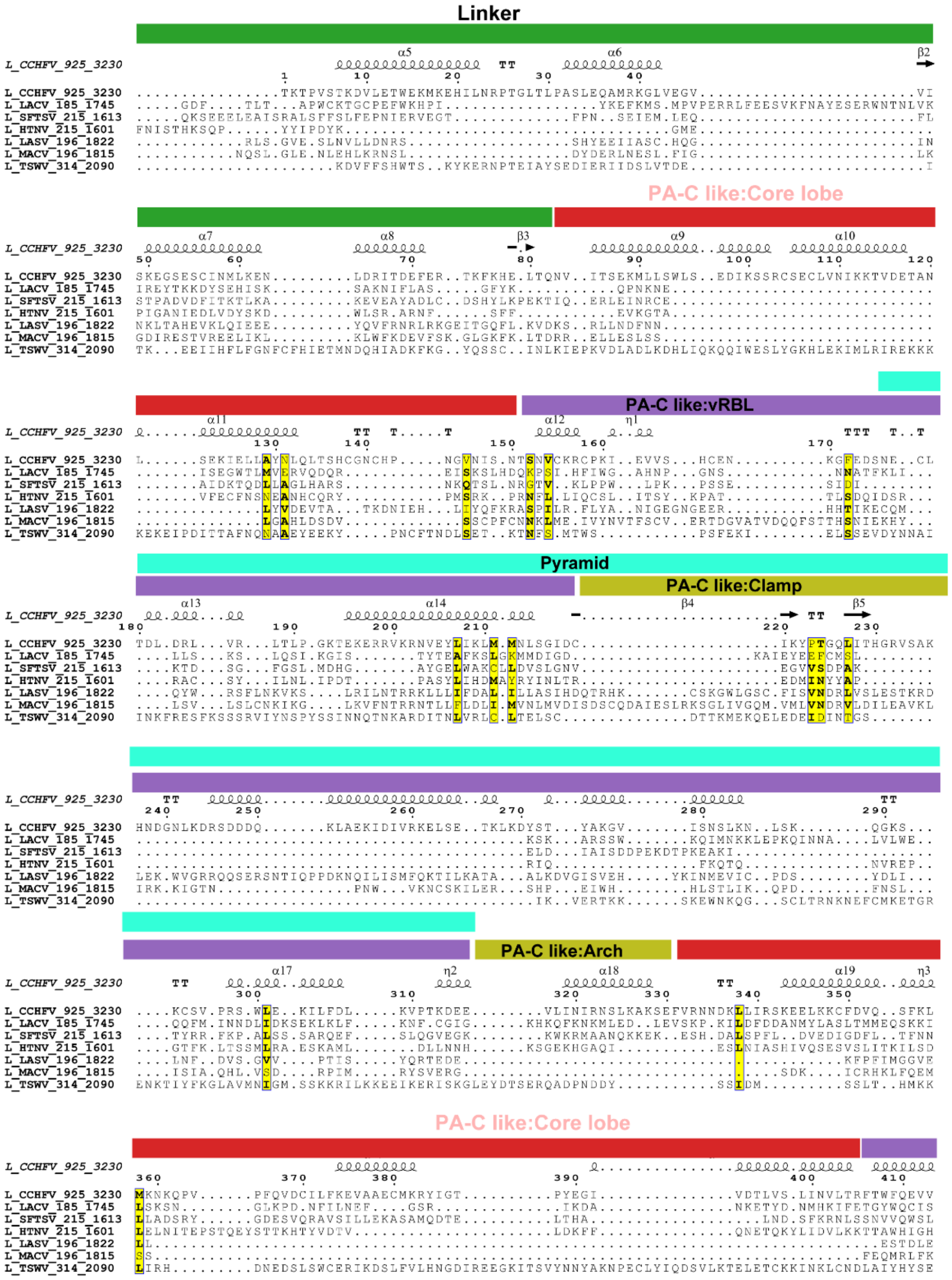
Linker
PA-C like:Core lobe
PA-C like:vRBL
Pyramid
PA-C like:Clamp
PA-C like:Arch
PA-C like:Core lobe
L_CCHFV_925_3230
L_LACV_185_1745
L_SFTSV_215_1613
L_HTNV_215_1601
L_LASV_196_1822
L_MACV_196_1815
L_TSWV_314_2090

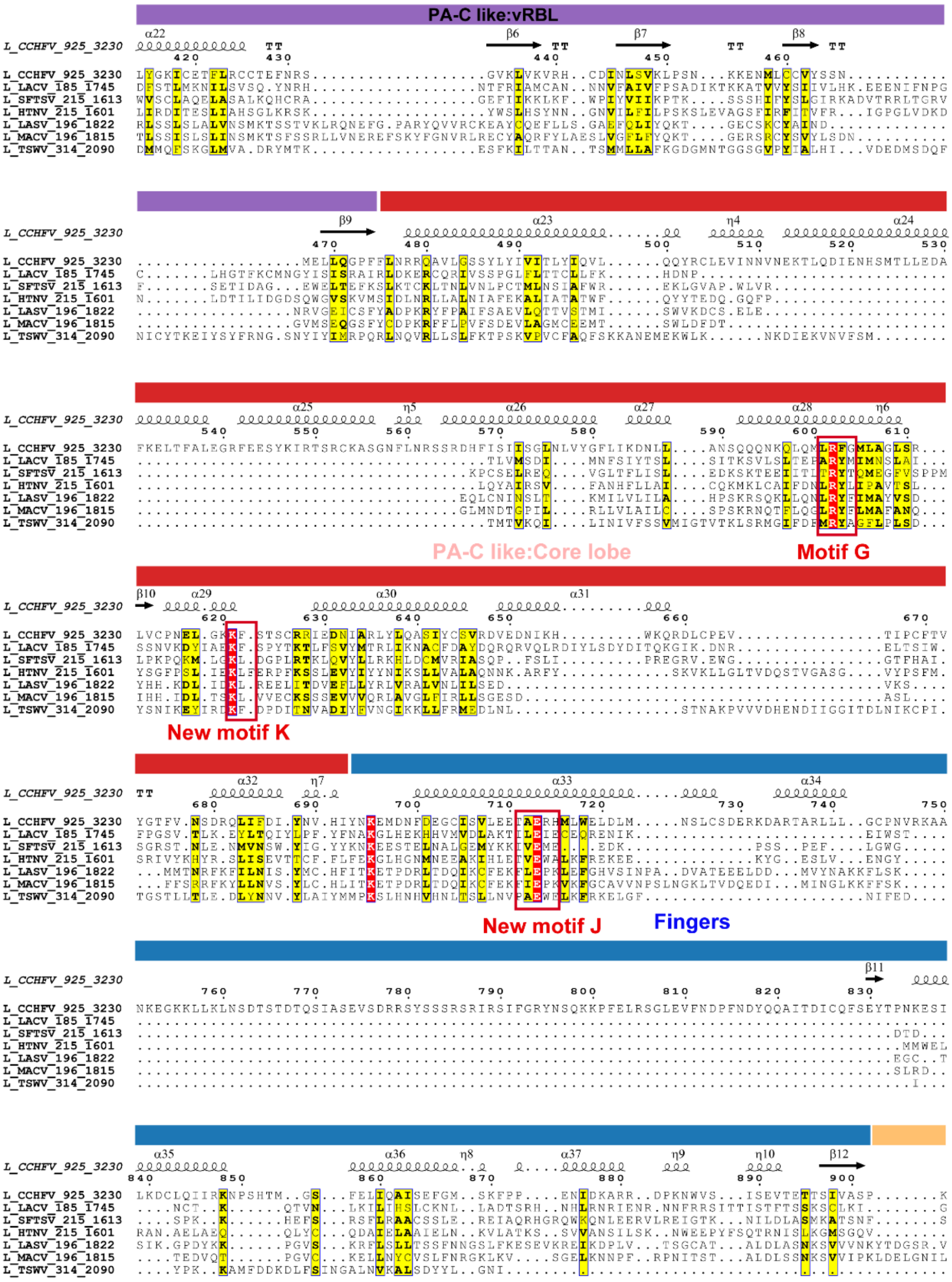
PA-C like:vRBL
PA-C like:Core lobe
Motif G
New motif K
New motif J
Fingers
L_CCHFV_925_3230
L_LACV_185_1745
L_SFTSV_215_1613
L_HTNV_215_1601
L_LASV_196_1822
L_MACV_196_1815
L_TSWV_314_2090

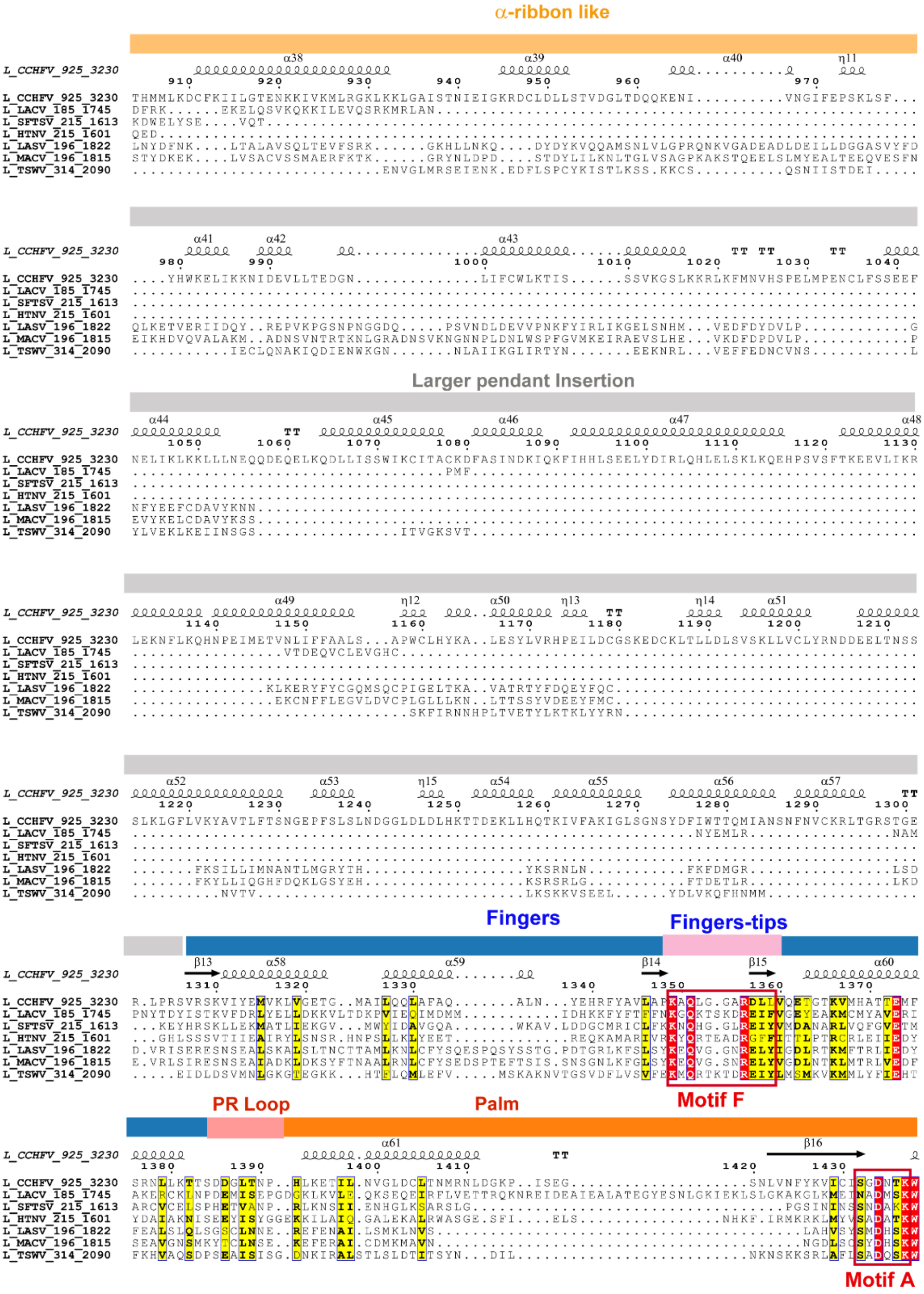
α-ribbon like
Larger pendant Insertion
Fingers
Fingers-tips
Motif F
PR Loop
Palm
Motif A
L_CCHFV_925_3230
L_LACV_185_1745
L_SFTSV_215_1613
L_HTNV_215_1601
L_LASV_196_1822
L_MACV_196_1815
L_TSWV_314_2090

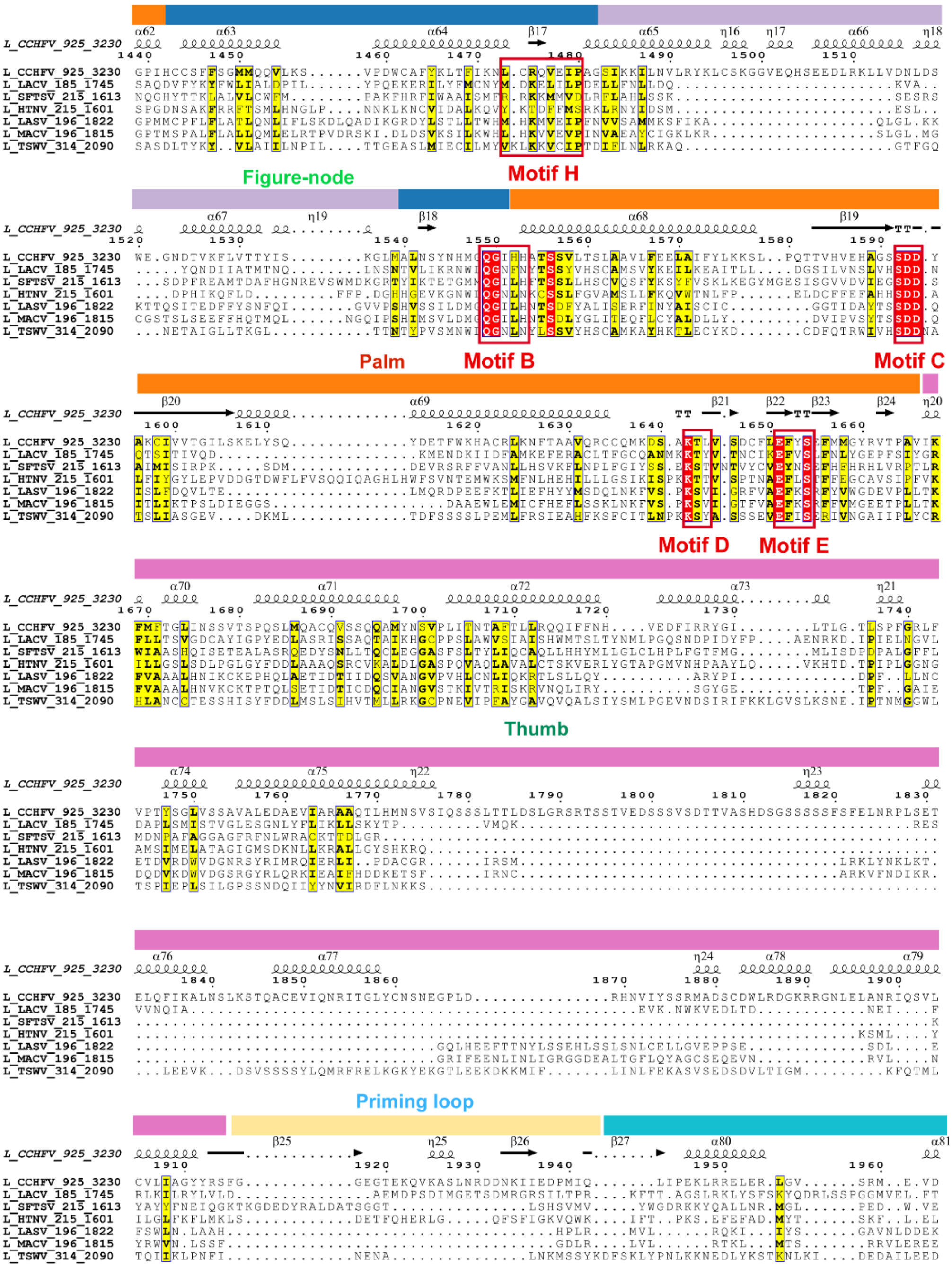
L_CCHFV_925_3230
L_LACV_185_1745
L_SFTSV_215_1613
L_HTNV_215_1601
L_LASV_196_1822
L_MACV_196_1815
L_TSWV_314_2090
Motif H
Figure-node
Palm
Motif B
Motif C
Motif D
Motif E
Thumb
Priming loop

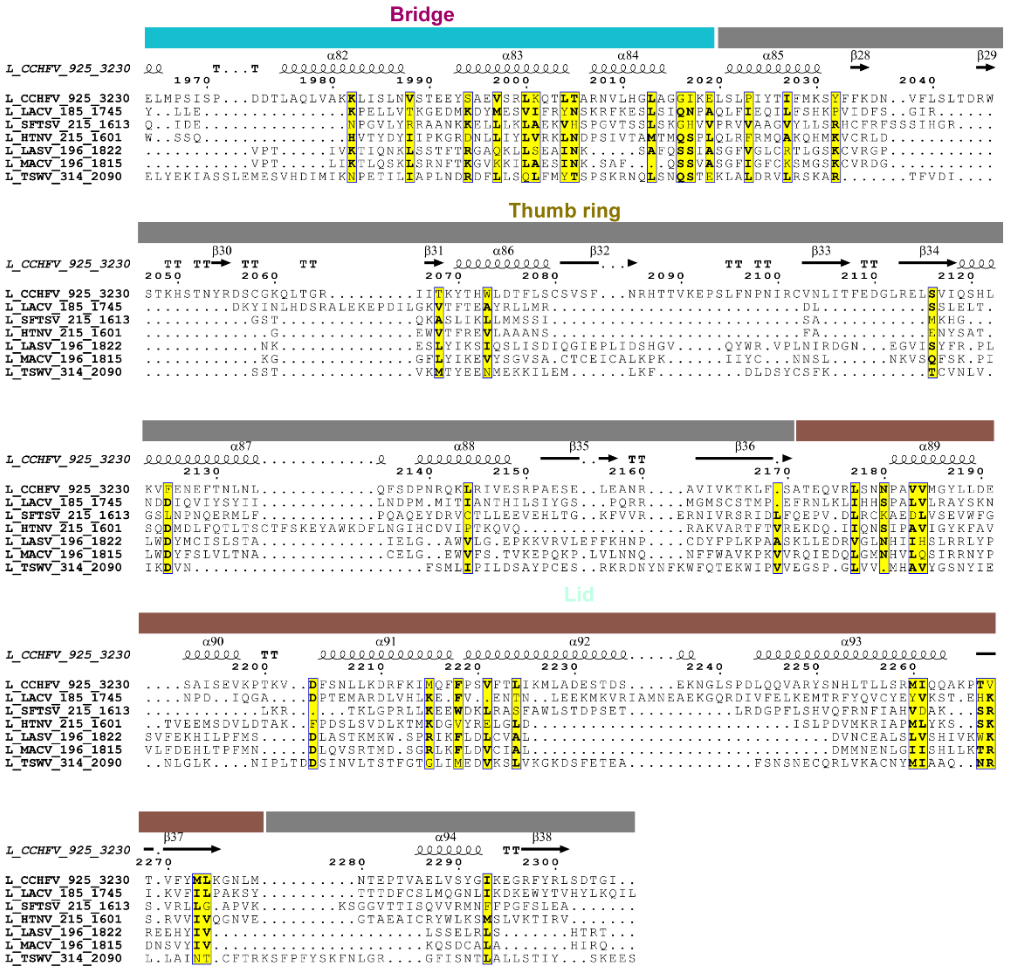

Figure S2 Structure-based sequence alignment of the polymerase core of CCHFV L with representative sNSVs.

Structure-based sequence alignment of the core region from CCHFV, LACV, SFTSV, HTNV, LASV, MACV and TSWV L proteins. Conserved polymerase motifs A-H are outlined with red boxes, and strictly or highly conserved residues are shaded in yellow. The secondary-structure elements of CCHFV L corresponding to the alignment are shown above the sequences. The domain organization of CCHFV L is indicated by colored bars and follows the domain definition presented in Fig. 1a.

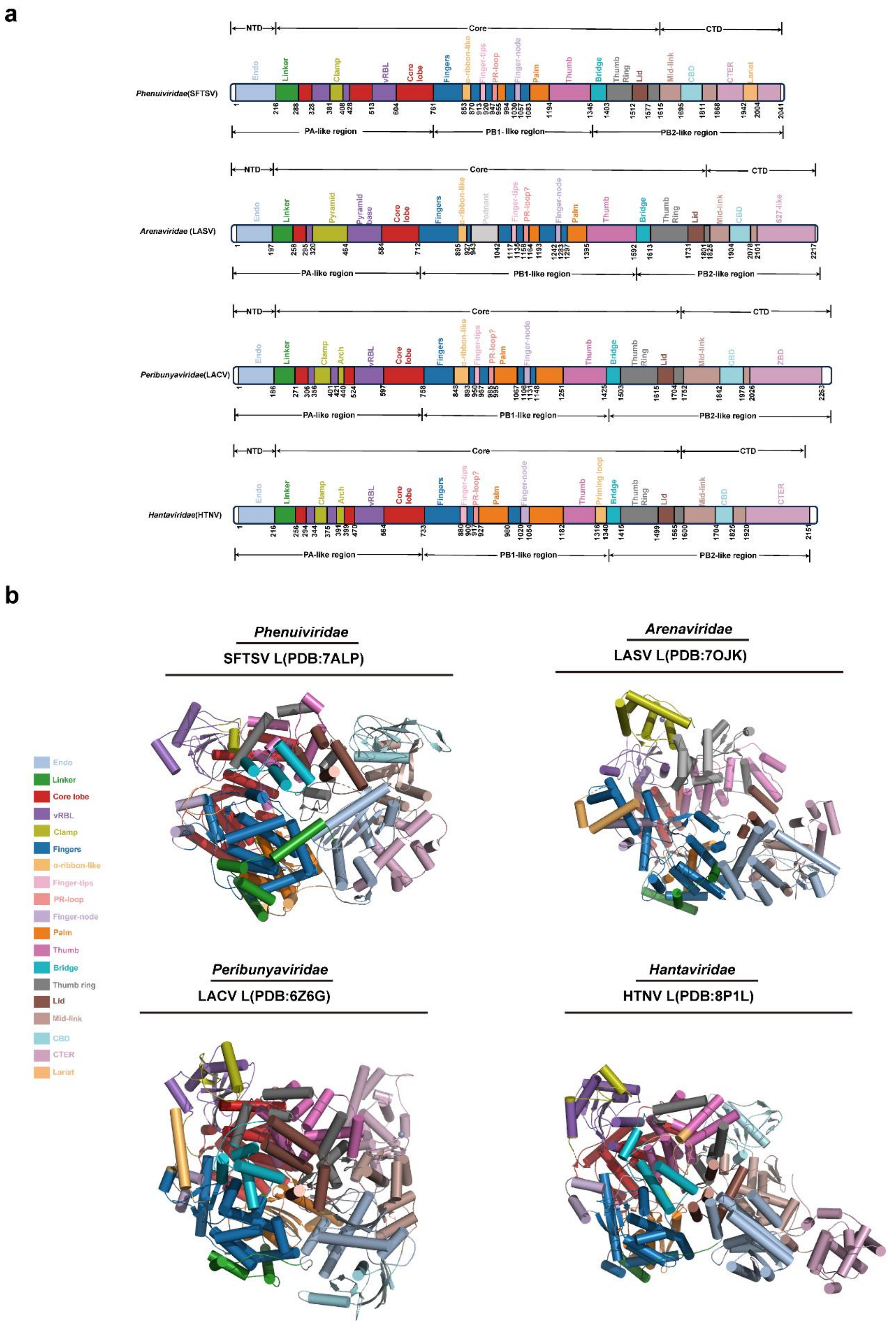

Figure S3 Comparison of domain organization and core architectures of representative *Bunyavirales* polymerases.

a. Schematic domain organization of L proteins from four representative *Bunyavirales* members: SFTSV (*Phenuiviridae*), LASV (*Arenaviridae*), LACV (*Peribunyaviridae*) and

HTNV (*Hantaviridae*). The PA-like, PB1-like (core) and PB2-like regions are indicated below each bar. Individual domains and structural elements are color-coded as in Fig. 1a, with residue boundaries labeled beneath the diagrams.

b. Ribbon representations of the L of SFTSV (PDB: 7ALP), LASV (PDB: 7OJK), LACV (PDB: 6Z6G) and HTNV (PDB: 8P1L), shown in the same color scheme as in panel (A).

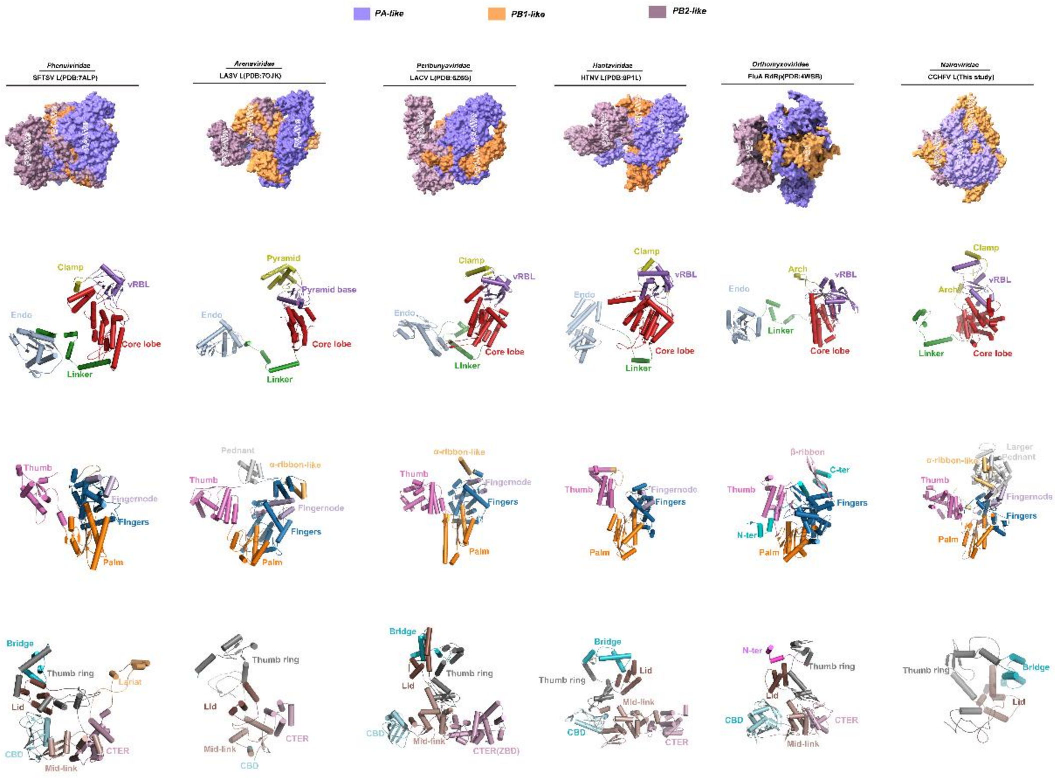

Figure S4 Comparative architecture of PA-like, PB1-like, and PB2-like regions in representative Bunyavirales polymerases and influenza A polymerase.

Upper: Surface and cartoon representations of L proteins from five *Bunyavirales* families: SFTSV (*Phenuiviridae*, PDB: 7ALP), LASV (*Arenaviridae*, PDB: 7OJK), LACV (*Peribunyaviridae*, PDB: 6Z6G) and HTNV (*Hantaviridae*, PDB: 8P1L) and CCHFV (*Nairoviridae*, this study), together with the influenza A virus polymerase (FluA, PDB: 4WSB) shown for reference. Lower: Cartoon representations highlighting the domain organization of each polymerase. The structures are partitioned into PA-like, PB1-like, and PB2-like regions, corresponding to the influenza polymerase subunits, to facilitate comparison of domain architecture across Bunyavirales polymerases.

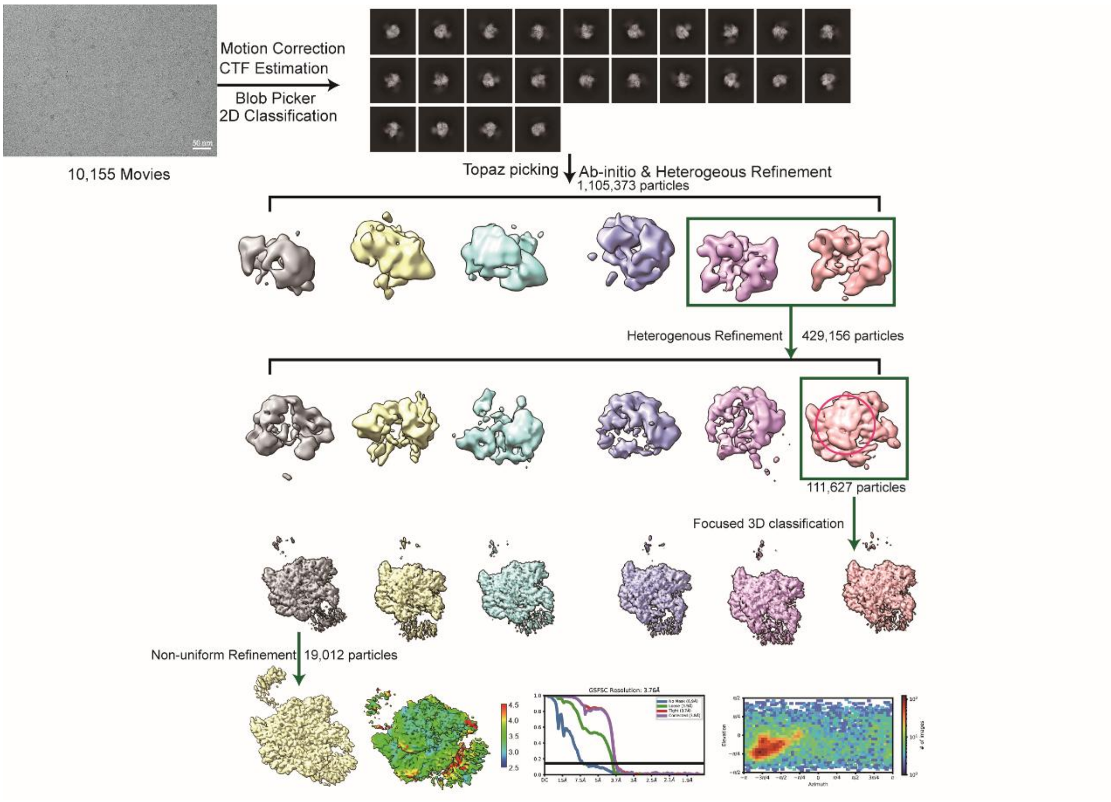

Figure S5 Data processing workflow of cryo-EM data analysis of 5′ hook vRNA bound CCHFV L.

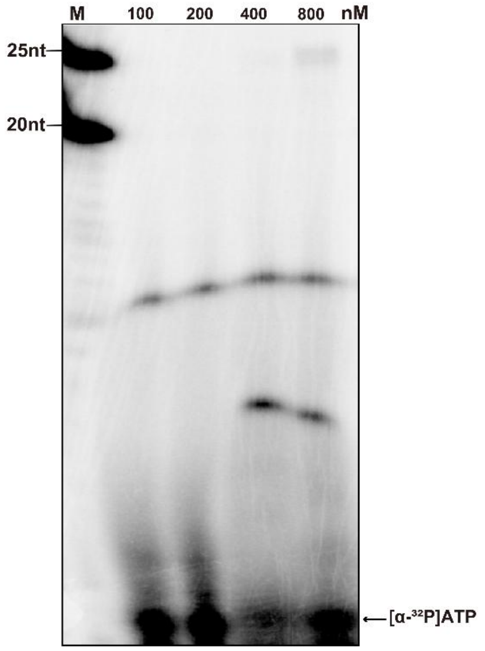

Figure S6 In vitro RNA synthesis activity of the core domain of the CCHFV L protein.

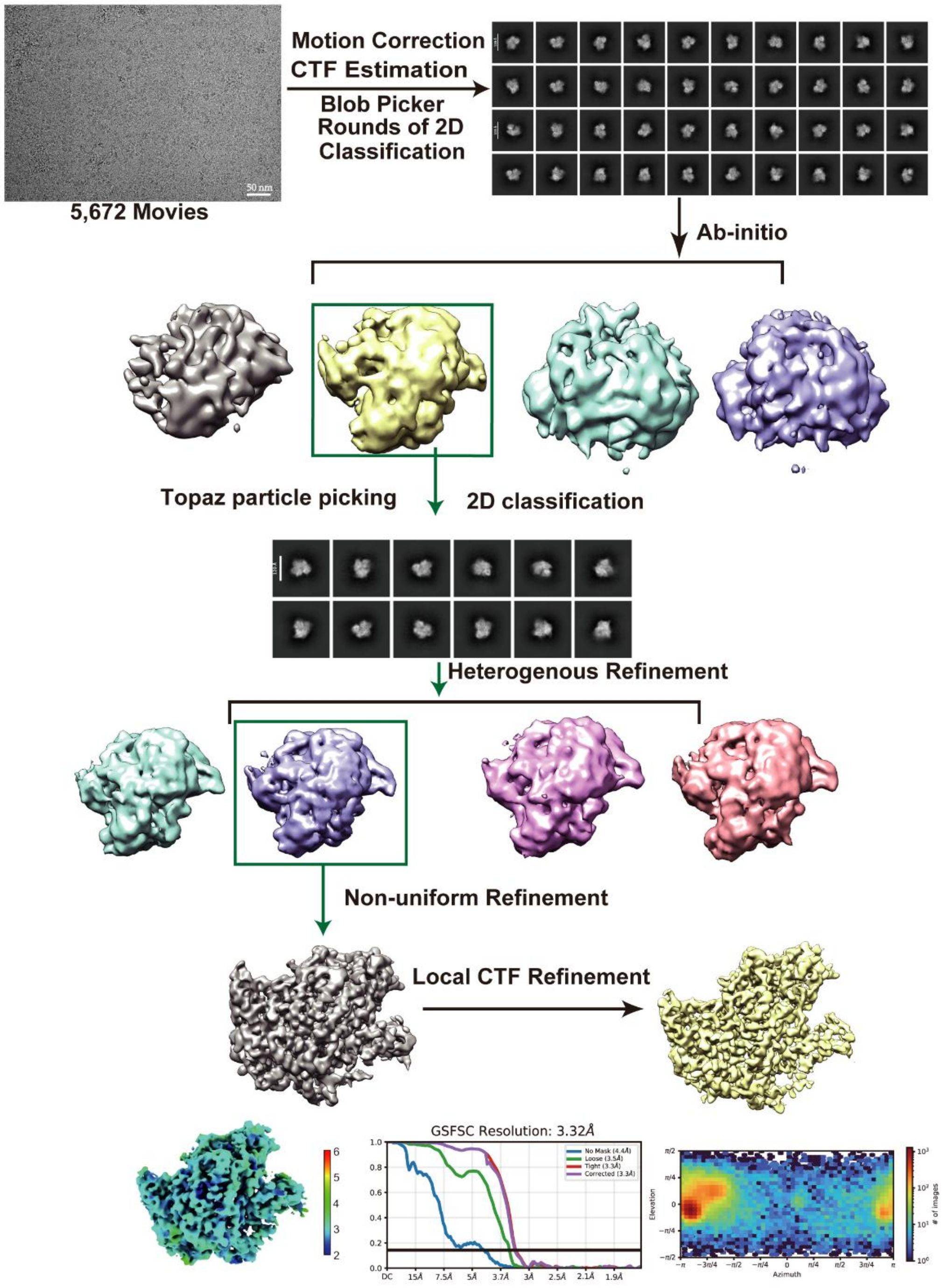

Figure S7 Data processing workflow of cryo-EM data analysis of 5′ hook vRNA bound CCHFV L Core.

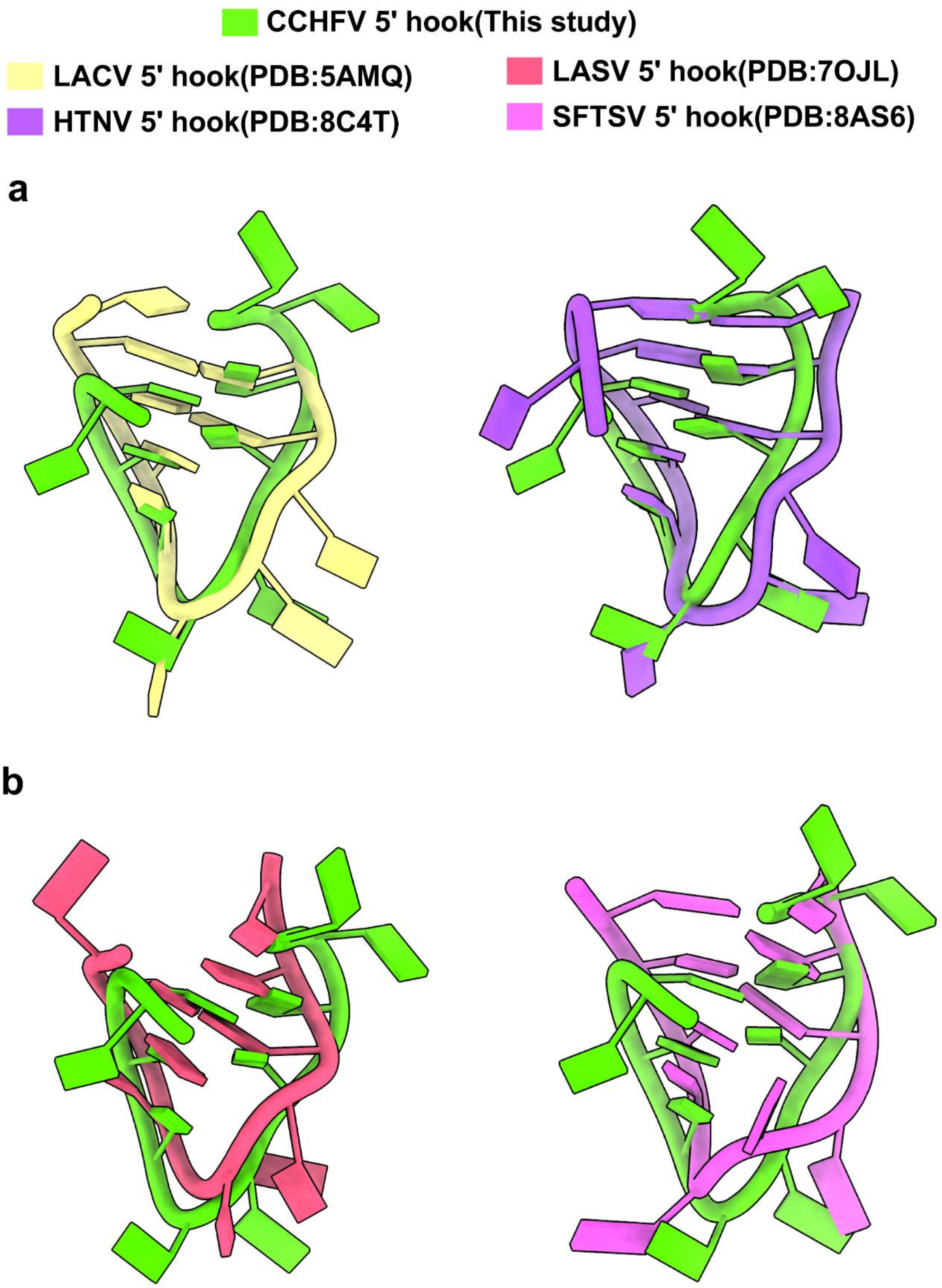

Figure S8 Comparison of 5′ vRNA hook structures from sNSVs.

a. Structural superposition of the CCHFV 5′ hook with the LASV (left) and SFTSV (right) hooks.

b. Structural superposition of the CCHFV 5′ hook with the LACV (left) and HTNV (right) hooks.

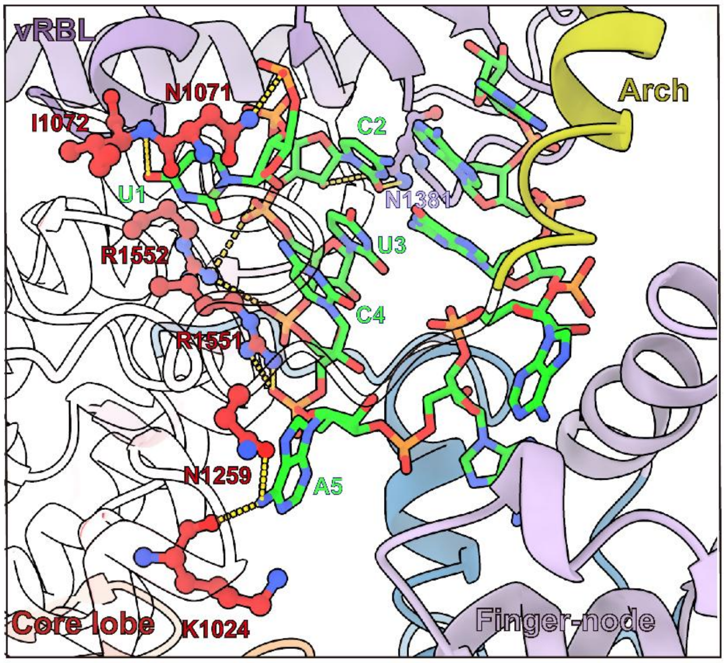

Figure S9 Detailed interactions between the CCHFV L polymerase and the first five nucleotides of the 5′ RNA hook.

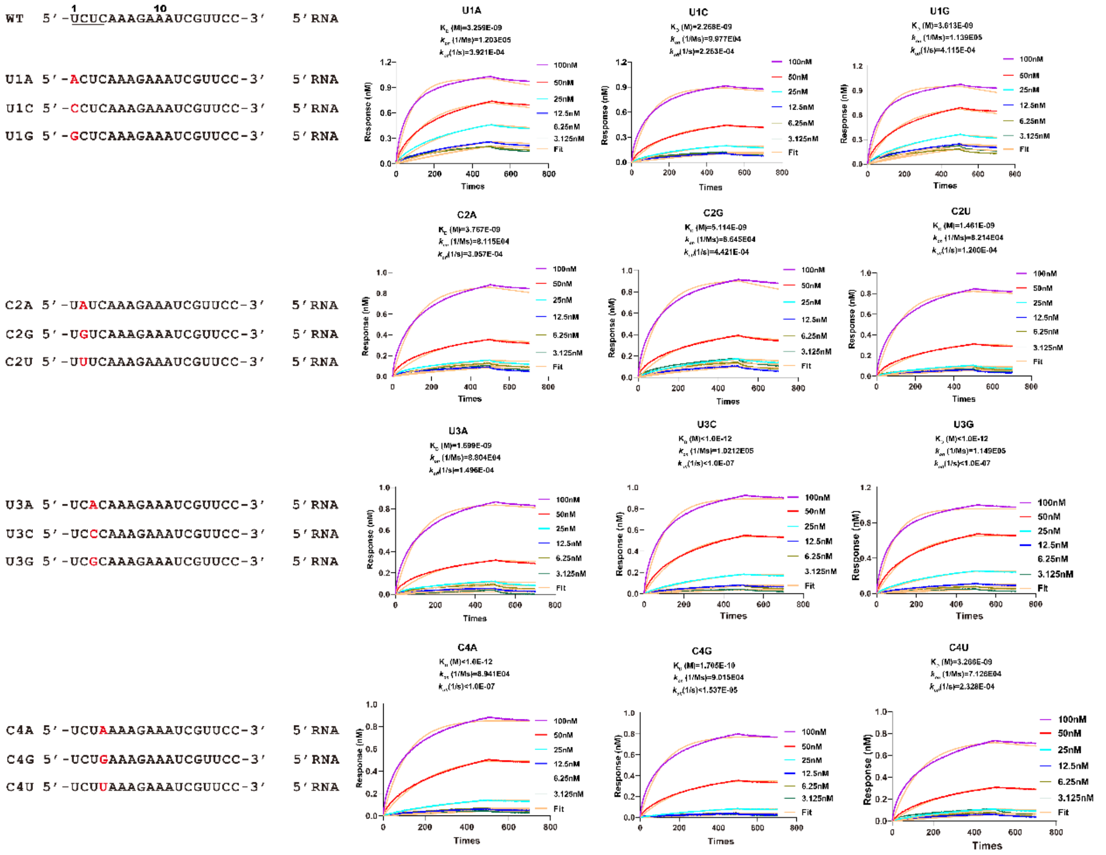

Figure S10 BLI analysis of CCHFV L binding to mutant 5′ RNA constructs.

BLI analysis for wild-type and mutant 5′ RNA constructs carrying single-nucleotide substitutions within the first four nucleotides. Mutated residues are highlighted in red. Most substitutions had little or modest effects on binding, whereas selected mutations enhanced binding affinity.

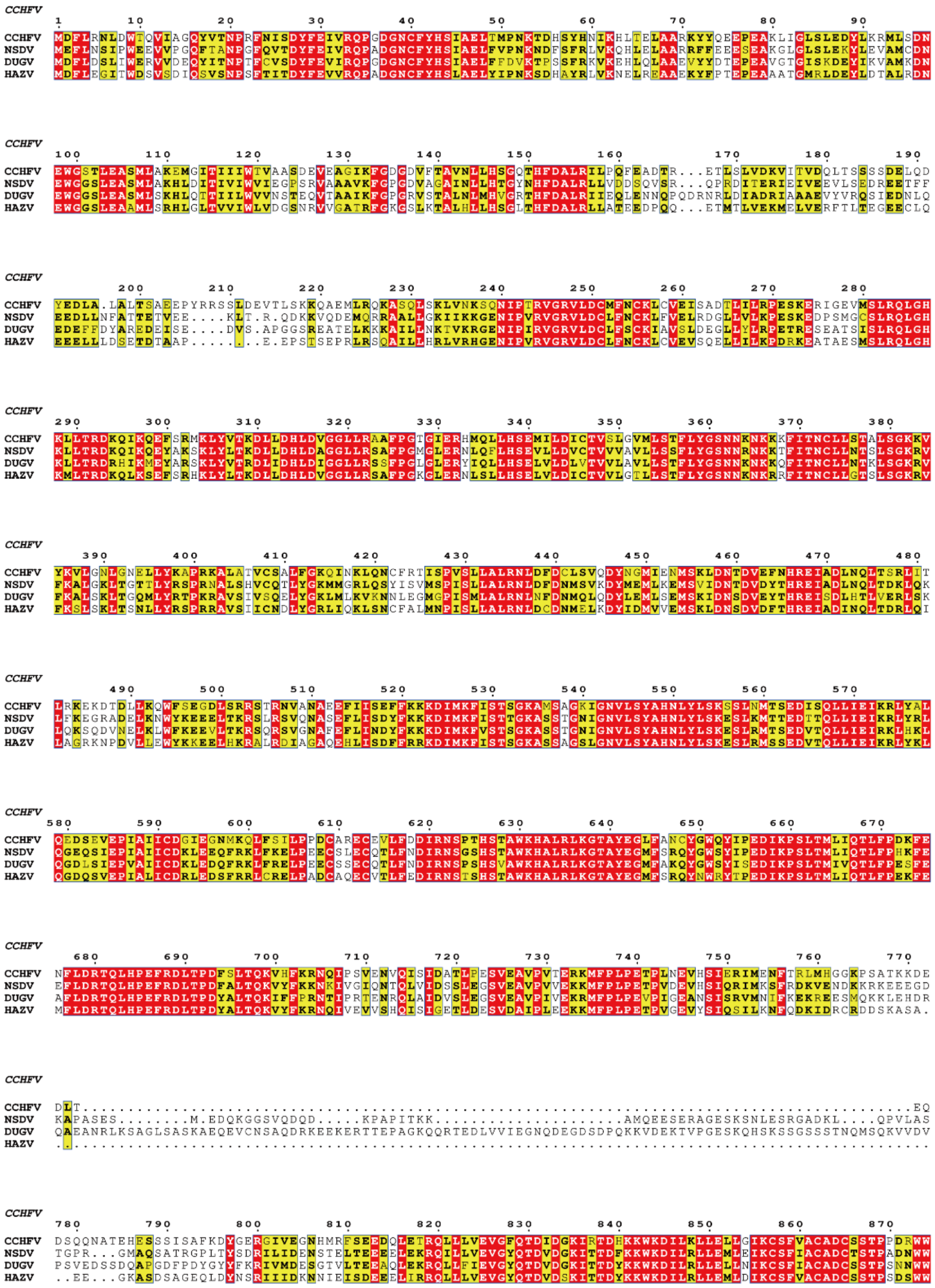
CCHFV
NSDV
DUGV
HAZV

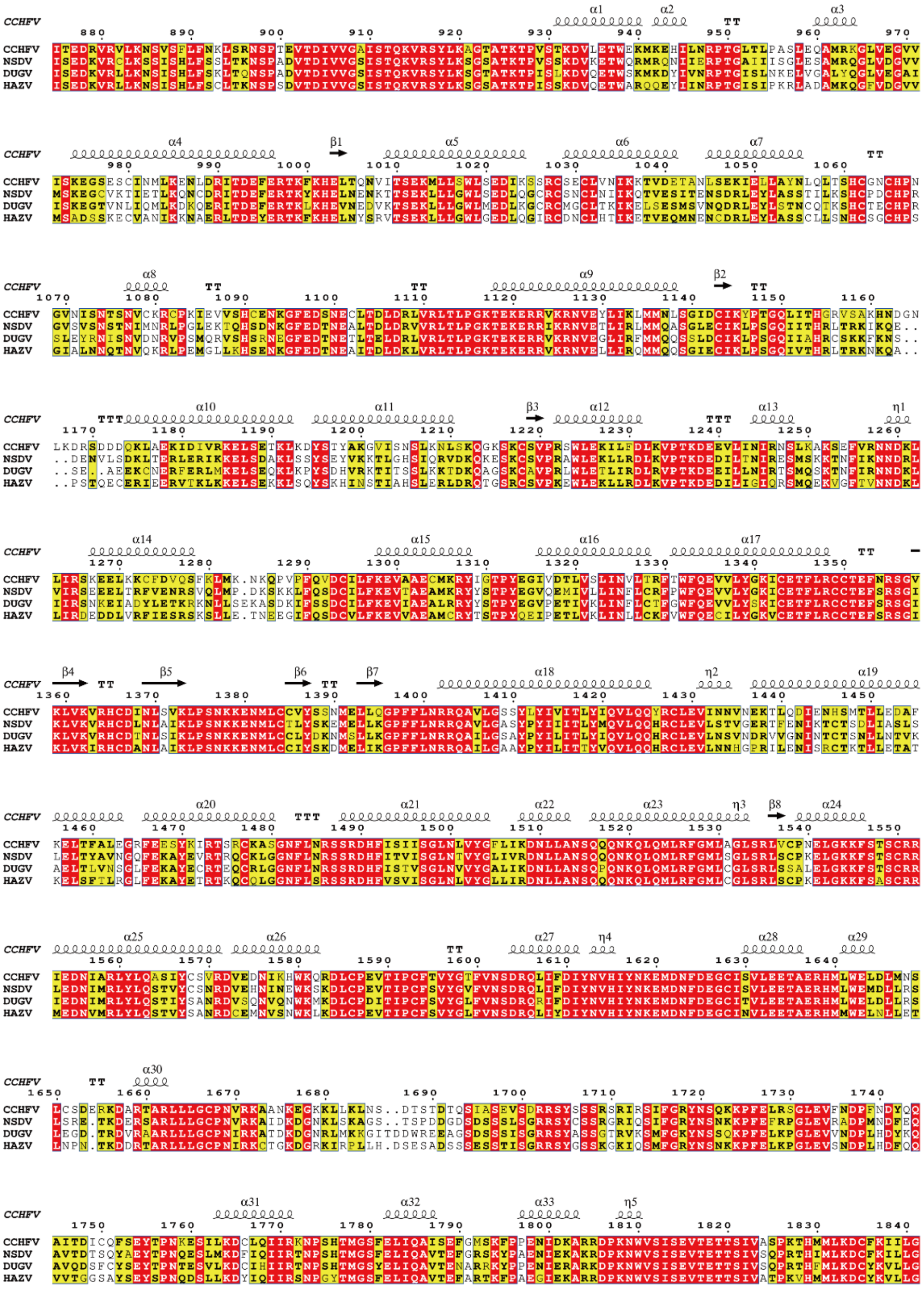
CCHFV
NSDV
DUGV
HAZV
α1 α2 α3 α4 α5 α6 α7 α8 α9 α10 α11 α12 α13 α14 α15 α16 α17 α18 α19 α20 α21 α22 α23 α24 α25 α26 α27 α28 α29 α30 α31 α32 α33
β1 β2 β3 β4 β5 β6 β7 β8
η1 η2 η3 η4 η5

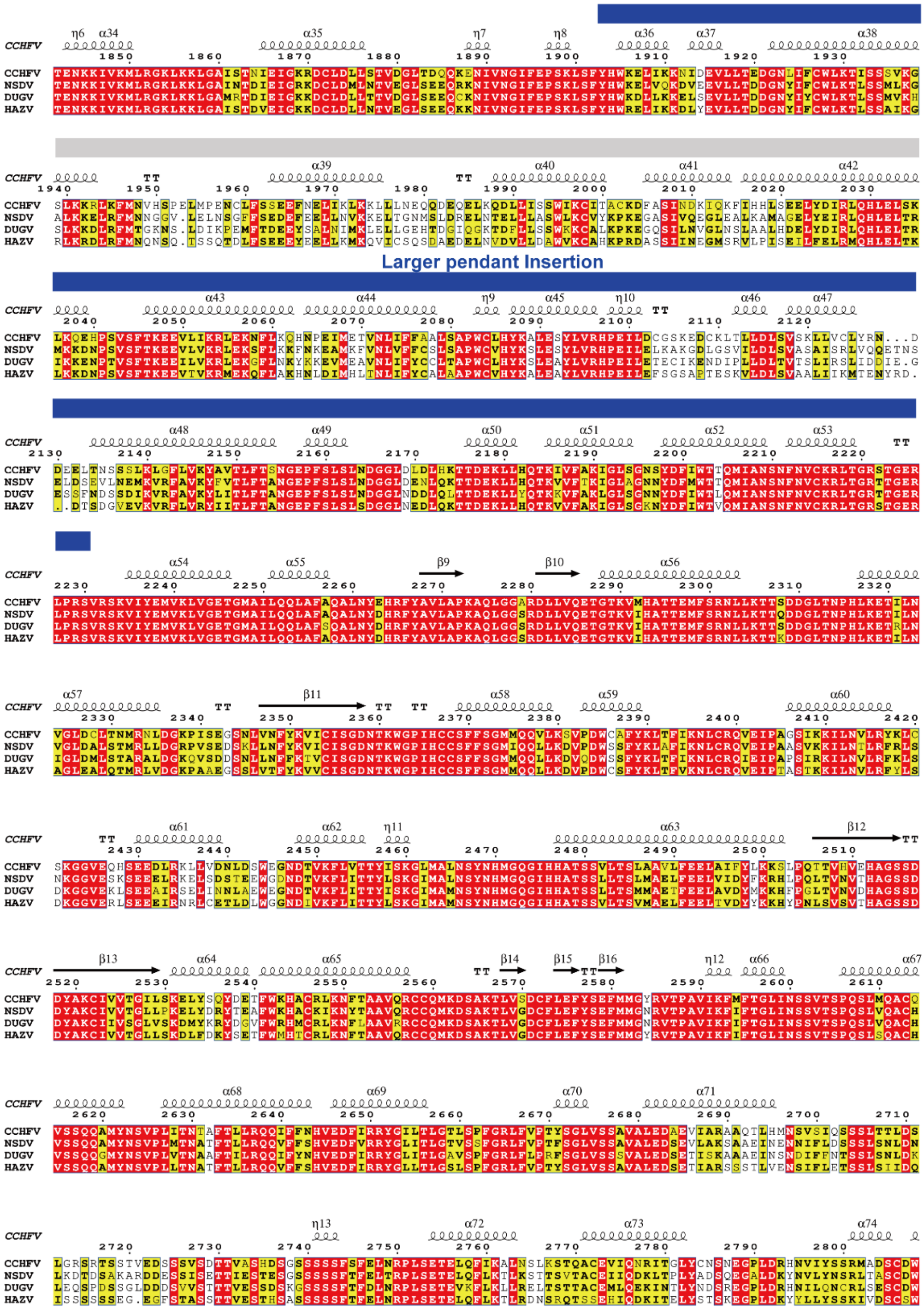
Larger pendant Insertion
CCHFV
NSDV
DUGV
HAZV

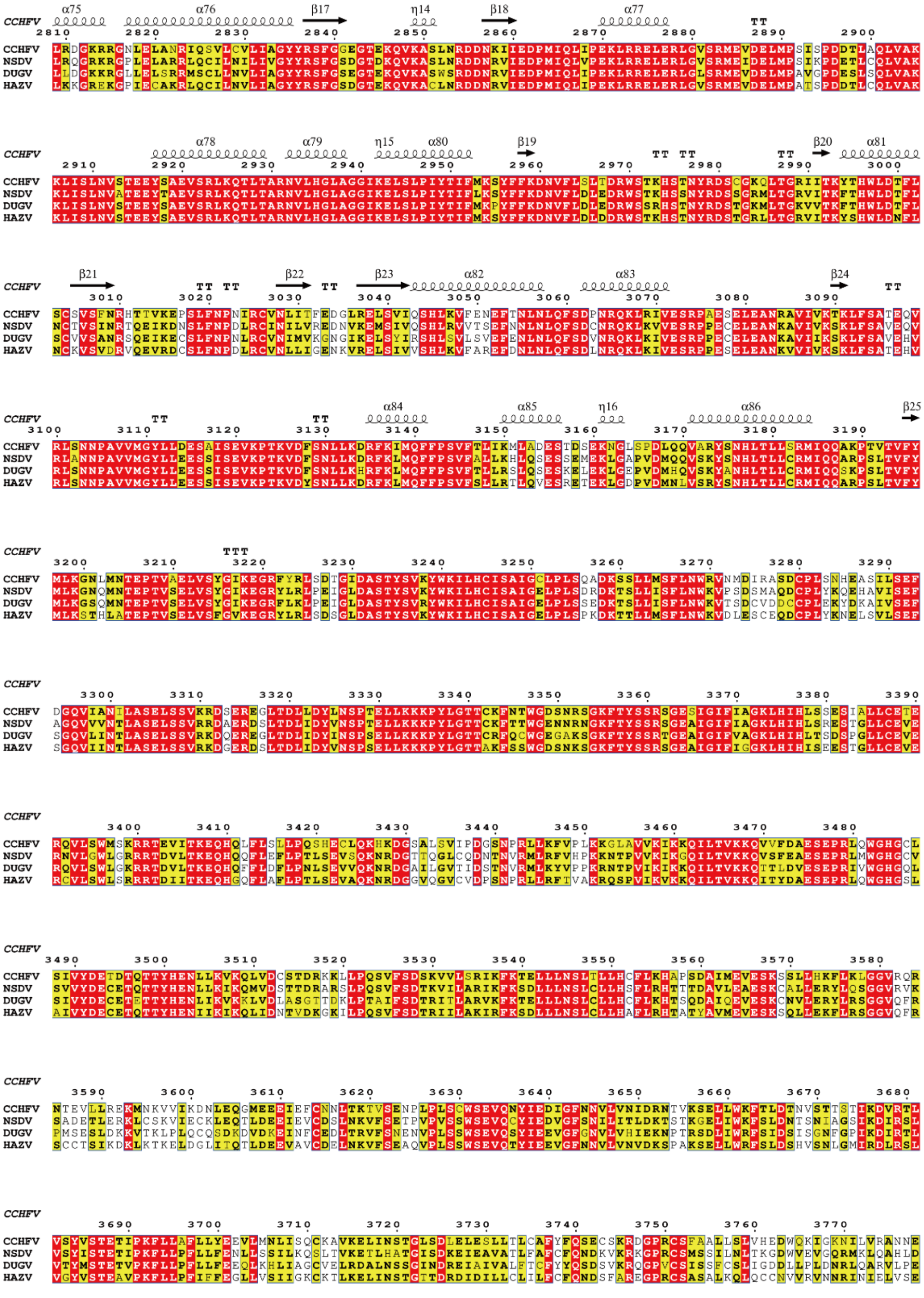

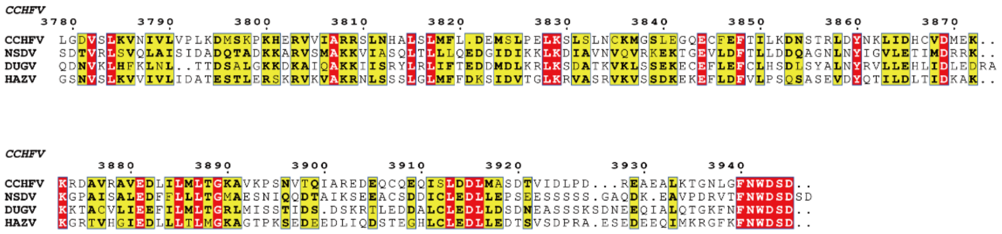

Figure S11 Structure-based sequence alignment of the CCHFV L protein with representative orthonairoviruses.

Structure-based sequence alignment of the L proteins from CCHFV, NSDV, DUGV, and HAZV. Conserved residues are outlined with red boxes, whereas strictly or highly conserved residues are highlighted in yellow. The secondary-structure elements of the CCHFV L protein corresponding to the aligned regions are shown above the sequences. CCHFV, Crimean–Congo hemorrhagic fever virus; NSDV, Nairobi sheep disease virus; DUGV, Dugbe virus; HAZV, Hazara virus.

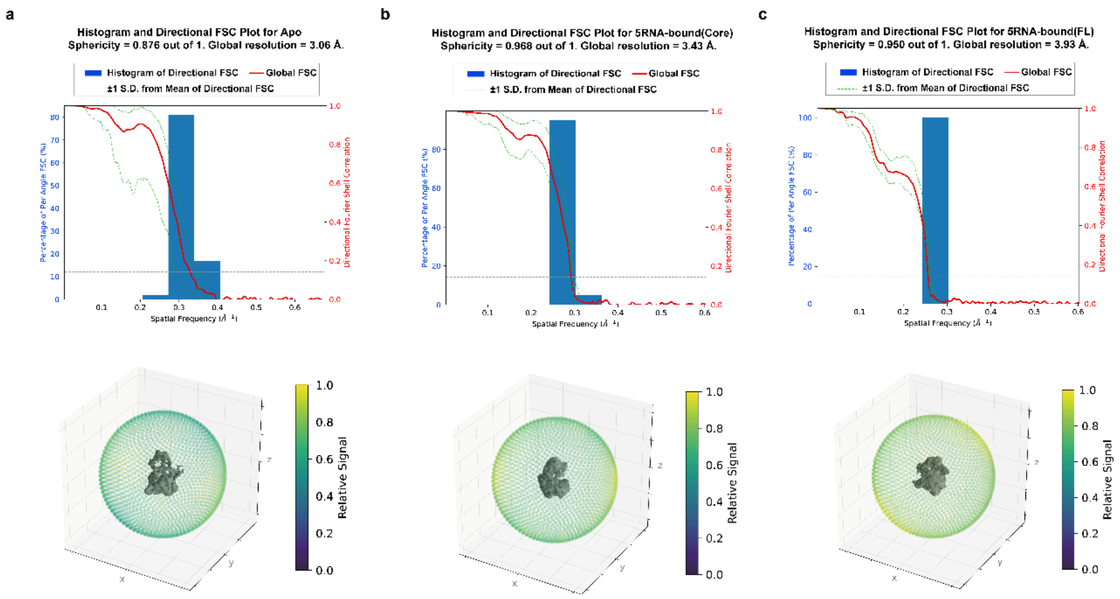

Figure S12 3D FSC analysis of cryo-EM reconstructions.

a-c: Upper: Directional FSC plots and histograms for the apo, 5′RNA-bound core, and 5′RNA-bound full-length reconstructions. Red lines indicate global FSC, and green dashed lines represent ±1 standard deviation of directional FSC. The sphericity values (0.876, 0.968, and 0.950) indicate largely isotropic reconstructions. Lower: Directional signal distributions mapped onto a sphere show broadly distributed signal across orientations, supporting sufficient angular sampling.

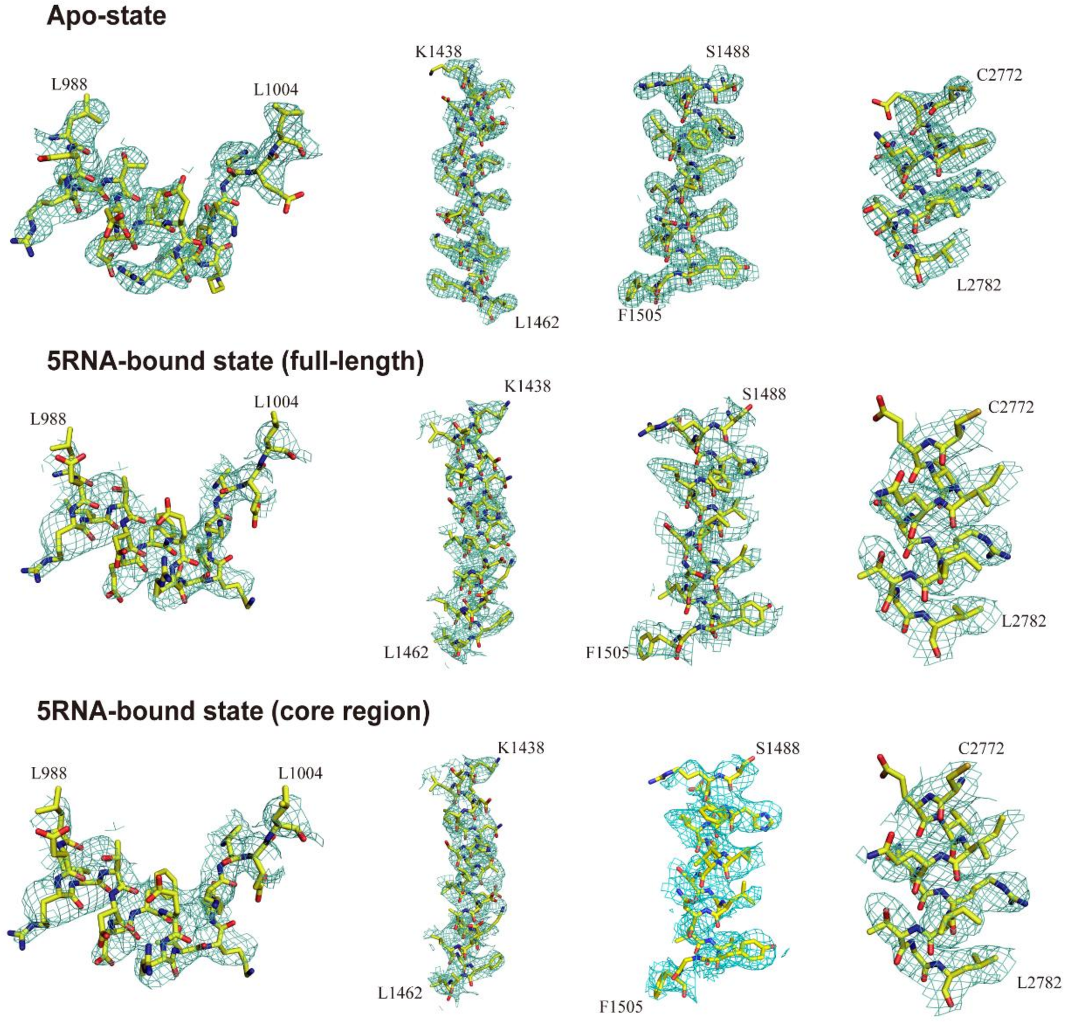

Figure S13 Representative EM densities for the different structures. The local map density for representative protein regions are indicated.

Supplementary Table S1 Cryo-EM data collection, refinement and validation statistics.

	Apo	5RNA-bound (FL)	5RNA-bound (Core)
**Data collection and processing**			
Magnification	165,000	105,000	105,000
Voltage (kV)	300	300	300
Election exposure ($e^-/Å^2$)	60	60	60
Defocus range (μm)	-1.5 to -2.0	-1.5 to -2.0	-1.5 to -2.0
Pixel size (Å)	0.74	0.83	0.83
Micrographs (no.)	9,040	11,010	5,672
Symmetry imposed	C1	C1	C1
Detector	Falcon4	K3	K3
Initial particle images (no.)	1,317,709	1,802,531	1,195,460
Final particle images (no.)	55,537	19,012	69,639
Map resolution (Å)	2.85	3.76	3.32
FSC threshold	0.143	0.143	0.143
**Refinement**			
Initial model used	Alphafold	Alphafold	Alphafold
Model resolution (Å)	1.8	3.6	2.1
FSC threshold	0.143	0.143	0.143
Map sharpening *B* factor ($Å^2$)	-74.3	-64.3	-94.1
Map Correlation Coefficient	0.69	0.59	0.72
Model composition			
Non-hydrogen atoms	12090	17243	11649
Protein residues	1511	2128	1431
Nucleotide	0	10	10
Ligands	0	0	0
Water	0	0	0
B factors ($Å^2$)			
Protein	149.78	248.89	83.25
Nucleotide	N/A	144.93	97.08
R.m.s deviations			
Bond lengths (Å)	0.004	0.004	0.004
Bond angles (°)	0.827	0.822	0.954
Validation			
MolProbity score	2.16	2.30	2.79
Clash score	10.77	20.23	18.73
Poor rotamers (%)	2.45	0.20	3.88
Ramachandran plot			

Disallowed (%)	0	0.8	0.5
Allowed (%)	4.52	7.58	10.63
Favored (%)	95.48	91.62	88.87

Supplementary Table S2

Synthesized genes in this study.

Gene names	genes sequence
His-CCHFV L-twin strepII	><br>ATGCATCATCACCATCACCATGGTACCATGGATTTCCTGAGAAACCTGGACT<br>GGACCCAAGTGATCGCCGGACAATACGTGACCAACCCTAGATTCAACATTT<br>CAGACTACTTTGAGATCGTGCGCCAGCCTGGAGATGGGAATTGCTTCTACC<br>ACTCTATCGCTGAGCTGACAATGCCGAACAAGACGGATCACAGCTACCATA<br>ACATCAAGCACCTGACAGAGTTGGCCGCTAGAAAATACTACCAGGAAGAA<br>CCTGAAGCCAAGCTGATTGGCCTGAGCCTAGAGGACTACCTGAAGAGGAT<br>GTTGTCGGACAATGAGTGGGGCAGCACCCTGGAGGCCAGCATGTTGGCAA<br>AGGAGATGGGCATCACCATCATCATCTGGACCGTGGCCGCCTCTGACGAGG<br>TGGAAGCCGGAATCAAGTTCGGCGACGGCGACGTGTTCACCGCCGTGAAC<br>CTGCTGCACAGCGGCCAGACACACTTTGATGCTCTGCGGATACTGCCTCAG<br>TTCGAAGCCGACACAAGAGAGACCCTTAGCCTGGTGGACAAGGTGATCAC<br>AGTGGACCAACTTACATCATCCTCTTCCGACGAACTGCAGGACTACGAGGA<br>TCTGGCCCTGGCCCTGACCAGCGCTGAGGAACCTTACCGGAGATCTAGCCT<br>GGACGAAGTGACCCTGTCGAAAAAGCAGGCTGAAATGCTGAGGCAGAAG<br>GCCAGCCAACTATCGAAACTGGTCAACAAGTCTCAGAACATCCCTACCAGA<br>GTGGGCAGGGTACTGGACTGCATGTTCAACTGCAAGCTGTGCGTGGAAATC<br>AGTGCTGACACACTGATCCTGAGACCCGAATCCAAGGAAAGAATCGGAGA<br>AGTGATGAGCCTCCGACAGCTCGGCCACAAGCTGCTGACCAGAGACAAGC<br>AGATCAAGCAGGAGTTCTCCAGAATGAAGCTGTACGTGACAAAGGACCTG<br>TTGGACCACCTCGACGTGGGCGGTCTGCTCAGAGCCGCATTCCCCGGCACA<br>GGCATCGAGAGACACATGCAGCTGCTGCACAGCGAAATGATCCTGGACATC<br>TGCACCGTTAGCCTGGGCGTGATGCTCAGCACCTTCCTGTACGGCTCCAAC<br>AACAAGAACAAGAAGAAGTTTATCACGAACTGCCTCCTGAGCACCGCCCT<br>GAGTGGAAAAAAGGTGTACAAGGTGCTGGGCAACCTGGGAAACGAACTG<br>CTGTACAAGGCCCCTAGAAAGGCTCTTGCCACTGTGTGCAGCGCCCTGTTC<br>GGCAAGCAGATTAACAAGCTGCAGAACTGTTTCAGAACCATCAGCCCTGT<br>GAGCCTGCTGGCCCTGAGAAACCTGGACTTTGATTGCCTGTCTGTGCAGGA<br>CTACAACGGCATGATCGAGAACATGAGCAAACTCGATAATACCGACGTGGA<br>GTTCAACCATAGAGAGATCGCAGATCTCAACCAGTTAACTAGCCGGCTTAT<br>CACCCTGAGAAAGGAGAAAGACACGGACCTGCTGAAACAATGGTTCTCGG<br>AAGGCGACCTGAGTCGGCGGTCTACCAGAAACGTGGCCAACGCCGAAGA<br>GTTTATCATCAGCGAGTTCTTCAAGAAGAAGGACATCATGAAATTCATTTCG<br>ACAAGCGGAAAGGCCATGAGCGCCGGTAAGATTGGCAACGTGCTGTCTTAT<br>GCCCACAACCTGTACCTGAGCAAATCTAGCCTGAACATGACAAGCGAAGA<br>CATCAGCCAGCTGCTTATCGAGATCAAGCGGCTATACGCTCTGCAGGAGGA

	CTCTGAGGTGGAACCTATCGCCATCATCTGCGATGGCATCGAGGGCAATATG<br>AAACAGCTGTTCAGTATCCTTCCTCCAGATTGTGCCAGGGAGTGCGAGGTA<br>CTGTTCGACGACATCAGAAATTCCCCTACCCACAGCACCGCCTGGAAACAC<br>GCCCTCAGACTGAAGGGCACCGCTTACGAGGGCCTGTTTGCCAACTGCTAC<br>GGCTGGCAGTATATCCCCGAGGACATTAAGCCCAGCCTGACCATGCTGATC<br>CAGACCTTATTTCCAGACAAGTTCGAGAATTTCCTGGACCGTACACAGCTG<br>CACCCCGAGTTCAGAGATCTGACCCCTGATTTCAGCCTCACGCAGAAGGTT<br>CACTTCAAGCGAAATCAGATACCGAGCGTTGAGAACGTCCAGATCAGCATC<br>GACGCCACACTTCCTGAATCCGTCGAAGCCGTGCCCGTGACAGAGCGGAA<br>GATGTTTCCACTGCCAGAGACTCCTCTGAACGAAGTTCATTCTATAGAAAG<br>GATCATGGAAAATTTCACACGTCTGATGCACGGCGGCAAGCCTTCAGCCAC<br>TAAGAAAGACGAGGACCTGACAGAGCAGGACAGCCAGCAGAACGCCACT<br>GAGCACGAGTCCAGCAGCATCTCAGCCTTCAAAGACTACGGCGAGCGGGG<br>CATCGTGGAGGGCAACCACATGCGGTTCAGCGAAGAGGATCAGCTGGAAA<br>CCCGGCAGCTCCTGCTGGTGGAAGTCGGCTTCCAGACAGACATTGACGGC<br>AAGATAAGAACCGACCACAAGAAGTGGAAGGACATCCTGAAACTCCTGGA<br>ACTCCTGGGGATAAAATGCTCGTTCGTGGCCTGTGCTGACTGCTCAAGCAC<br>ACCGCCTGATAGATGGTGGATCACAGAGGACAGAGTGCGGGTGCTTAAGA<br>ACTCCGTGAGCTTCCTGTTCAACAAGCTGAGCAGAAACTCTCCCACCGAG<br>GTGACAGATATCGTGGTCGGAGCCATCAGCACACAAAAGGTGCGCTCTTAC<br>CTGAAAGCGGGCACCGCTACCAAGACACCTGTGTCCACCAAGGACGTGTT<br>GGAAACCTGGGAAAAGATGAAGGAGCACATCCTGAACAGACCTACCGGCC<br>TGACACTGCCTGCTTCCCTGGAACAGGCCATGAGAAAGGGCCTGGTGGAA<br>GGCGTGGTGATCAGCAAGGAAGGCAGCGAGAGCTGCATCAATATGCTCAA<br>GGAGAATCTGGATAGAATAACCGACGAGTTCGAGAGAACCAAGTTCAAAC<br>ATGAGCTGACACAGAATGTTATAACCTCCGAGAAGATGCTGCTGAGCTGGC<br>TGAGCGAAGATATCAAGAGCTCGCGGTGTAGCGAGTGCCTGGTGAACATCA<br>AGAAGACTGTGGACGAGACAGCCAATCTGTCCGAAAAGATCGAGCTGCTG<br>GCATATAACCTTCAGCTGACCTCCCACTGCGGCAACTGCCACCCTAATGGC<br>GTGAACATCAGTAACACCTCCAACGTATGCAAGAGATGTCCTAAAATCGAG<br>GTGGTGAGTCACTGCGAAAACAAAGGCTTCGAGGATTCTAATGAGTGCCT<br>GACCGACTTAGACAGACTGGTGCGGCTGACACTGCCCGGCAAGACAGAGA<br>AAGAGCGGCGGGTGAAGCGAAACGTGGAATACCTGATCAAGCTGATGATG<br>AACCTGTCCGGAATCGACTGTATCAAGTACCCCACCGGCCAGCTGATCACT<br>CACGGCAGAGTGAGCGCCAAGCATAATGACGGCAACCTCAAGGATCGGTC<br>CGATGACGATCAGAAGCTGGCCGAGAAGATCGACATCGTCAGAAAGGAGC<br>TGTCCGAGACAAAATTGAAGGACTACAGCACATACGCCAAGGGAGTGATC<br>AGCAACTCTCTGAAGAACCTGTCTAAGCAGGGCAAGTCCAAGTGCTCCGT<br>GCCCAGAAGCTGGTTGGAGAAGATCCTGTTCGACCTGAAGGTGCCAACGA<br>AGGACGAAGAAGTGCTGATCAATATCAGAAACAGCCTTAAGGCCAAATCTG<br>AGTTTGTGAGAAACAACGACAAGCTGCTGATCAGGTCCAAGGAGGAGCTG<br>AAGAAGTGCTTCGACGTGCAGTCCTTCAAACTCATGAAGAACAAGCAACC<br>TGTACCTTTTCAGGTGGACTGCATCCTCTTCAAAGAGGTGGCCGCTGAGTG<br>CATGAAGAGATACATCGGCACTCCCTACGAGGGCATCGTGGACACCCTGGT

	CTCCCTGATCAACGTGCTGACTAGATTCACCTGGTTCCAAGAAGTCGTGCT<br>GTACGGAAAAATCTGCGAAACATTTCTGCGGTGTTGTACCGAGTTCAACAG<br>AAGCGGCGTTAAGCTGGTGAAGGTGCGGCACTGTGATATCAACCTGTCCGT<br>GAAACTGCCTAGCAACAAGAAGGAAAACATGCTGTGCTGCGTGTACAGCA<br>GCAACATGGAACTGTTGCAGGGCCCTTTTTCCTCAATAGAAGACAAGCCG<br>TGCTCGGCAGCAGCTACCTGTACATCGTGATCACCCTCTATATCCAGGTGCT<br>CCAGCAGTACAGATGCCTGGAGGTGATCAACAATGTGAACGAGAAGACAC<br>TGCAGGACATAGAGAATCACTCCATGACCCTGCTGGAAGACGCCTTTAAAG<br>AGCTGACCTTCGCCCTGGAAGGAAGATTCGAAGAGTCTTACAAGATTAGAA<br>CCTCCCGCTGTAAAGCTAGCGGCAACTTCCTGAACAGAAGCAGCAGAGAC<br>CACTTCATCTCAATCATCTCTGGACTGAATCTGGTGTATGGCTTCCTGATCA<br>AGGACAATCTCCTGGCCAACTCGCAGCAGCAGAACAAGCAGCTGCAGATG<br>CTAAGATTCGGCATGCTGGCTGGCCTGAGCAGGTTGGTTTGCCCGAACGAG<br>TTGGGCAAGAAGTTCAGCACATCCTGCAGACGGATCGAGGACAACATCGC<br>TAGACTGTACCTGCAAGCCAGCATTTACTGCAGCGTTAGGGACGTGGAGGA<br>TAACATTAAACACTGGAAGCAAAGAGATCTGTGCCCTGAGGTGACGATCCC<br>TTGTTTCACCGTCTACGGCACCTTCGTCAACAGTGACAGACAGCTGATTTT<br>CGACATCTATAACGTGCACATCTACAACAAGGAAATGGACAATTTCGACGA<br>GGGTTGTATCAGCGTGCTCGAAGAGACAGCCGAGAGACACATGCTGTGGG<br>AGCTGGATCTGATGAACAGCCTGTGCTCCGATGAACGGAAGGATGCCAGA<br>ACCGCCCGTCTGCTGCTTGGATGTCCAAATGTGCGGAAGGCCGCCAACAA<br>GGAGGGCAAGAAGCTGTTAAAGCTGAACAGTGATACCTCAACCGACACCC<br>AGAGCATCGCCAGCGAGGTGTCTGATAGAAGAAGTTATTCTTCATCTAGATC<br>TCGGATCAGGTCTATCTTCGGCAGATACAACAGCCAGAAAAAACCTTTCGA<br>GCTGAGATCCGGCCTGGAGGTTTTCAACGACCCTTTTAACGATTACCAGCA<br>GGCCATCACCGATATCTGTCAGTTTAGCGAATACACCCCTAATAAAGAGTCT<br>ATCCTGAAGGATTGCCTGCAGATCATCAGAAAGAACCCCTCCCACACCATG<br>GGCAGCTTTGAGTTGATCCAGGCCATTAGCGAGTTTGGCATGAGCAAGTTC<br>CCTCCTGAAAACATCGACAAGGCCCGGCGGGACCCCAAGAACTGGGTGTC<br>CATCTCAGAAGTGACCGAAACGACCAGCATCGTGGCTAGCCCCAAGACCC<br>ACATGATGCTGAAGGATTGCTTTAAAATCATCCTGGGAACCGAAAACAAAA<br>AGATCGTCAAGATGCTGAGAGGCAAACTGAAAAAGCTGGGCGCCATCTCT<br>ACCAACATCGAGATCGGGAAGCGGGATTGTCTGGATCTGCTGAGTACAGTC<br>GACGGCCTGACCGACCAGCAGAAAGAAAACATCGTGAACGGCATCTTCGA<br>GCCCAGCAAGCTGAGCTTCTACCACTGGAAGGAGCTGATCAAAAAGAACA<br>TCGACGAGGTGCTGCTGACAGAGGATGGGAACCTGATCTTCTGCTGGCTGA<br>AGACCATTAGCAGCTCTGTGAAGGGCAGCCTGAAGAAGCGGCTGAAATTT<br>ATGAACGTTCACTCTCCAGAACTGATGCCTGAAAACTGCCTGTTCAGCTCA<br>GAAGAGTTCAACGAATTAATCAAACTGAAGAAGCTGCTCCTGAATGAGCA<br>GCAGGATGAGCAGGAACTGAAGCAGGATCTGCTGATCAGCTCCTGGATCA<br>AGTGCATCACCGCTTGCAAGGACTTCGCCTCCATCAATGACAAGATCCAGA<br>AGTTTATCCACCACCTGTCCGAGGAACTGTACGACATCCGGCTGCAACACC<br>TGGAGCTCAGCAAACTGAAGCAGGAGCACCCTTCTGTGTCTTTCACCAAA<br>GAGGAGGTGCTCATCAAGAGATTGGAGAAGAACTTCCTGAAACAGCACAA

	CCCTGAAATAATGGAAACAGTCAATCTGATCTTTTTCGCTGCCCTGAGCGCT<br>CCTTGGTGTCTGCACTACAAGGCCCTGGAAAGTTACCTGGTCAGACATCCC<br>GAGATTCTCGACTGTGGCTCCAAAGAAGATTGCAAGCTGACACTGCTGGAT<br>CTGTCTGTGAGCAAGCTGCTGGTGTGCCTGTACCGGAACGACGATGAAGA<br>GCTCACGAATAGCTCCTCCCTCAAACTGGGCTTCTTGGTGAAGTACGCCGT<br>GACCCTGTTCACTAGCAACGGCGAGCCTTTTAGCCTGAGCCTGAACGACGG<br>CGGCTTGGATCTGGACCTCCATAAGACCACAGACGAGAAACTCCTGCACC<br>AGACAAAGATCGTGTTCGCCAAAATTGGACTCTCAGGCAACTCATACGACT<br>TCATCTGGACCACACAGATGATCGCCAACTCAAATTTCAACGTGTGCAAAA<br>GGCTGACCGGCAGAAGCACCGGAGAGAGGCTGCCAAGAAGCGTGAGATC<br>TAAAGTGATCTACGAGATGGTGAAGCTGGTGGGCGAAACTGGAATGGCTAT<br>CCTTCAGCAACTGGCCTTCGCCCAGGCGCTCAATTACGAACACCGGTTCTA<br>TGCTGTTCTCGCACCTAAGGCACAGCTTGGTGGCGCCAGAGATCTGTTAGT<br>GCAGGAGACAGGCACCAAGGTTATGCACGCCACCACCGAGATGTTCAGCA<br>GAAATCTGCTGAAGACCACATCCGATGATGGCTTAACCAACCCTCACCTGA<br>AGGAAACCATCCTGAACGTGGGCCTCGACTGCCTGACCAACATGCGGAAC<br>CTGGACGGCAAGCCAATCAGCGAAGGAAGTAACCTGGTGAACTTCTATAA<br>GGTGATCTGCATCTCAGGAGATAACACCAAATGGGGCCCCATCCACTGCTG<br>CTCTTTCTTCAGCGGCATGATGCAGCAGGTGTTGAAGTCTGTGCCCGATTG<br>GTGCGCCTTCTACAAACTGACATTCATCAAGAACCTCTGCCGGCAGGTGGA<br>GATCCCCGCCGGCAGCATCAAAAAGATCCTGAACGTGCTGCGGTACAAGCT<br>GTGCTCCAAGGGCGGAGTGGAGCAACACAGCGAAGAGGACCTGAGAAAG<br>TTGCTGGTGGACAACCTGGACTCCTGGGAGGGTAACGACACCGTGAAATT<br>CCTGGTCACCACGTACATCAGCAAGGGACTAATGGCCCTGAACTCTTACAA<br>TCACATGGGCCAGGGCATCCACCACGCTACCAGCAGCGTGCTGACCAGTCT<br>GGCCGCTGTCCTGTTCGAGGAGCTGGCCATTTTCTACCTGAAAAAGTCCTT<br>GCCGCAGACCACCGTTCATGTGGAACACGCCGGATCGTCCGACGACTACG<br>CCAAGTGCATCGTGGTCACTGGCATCCTGTCCAAGGAACTGTACTCCCAAT<br>ACGACGAGACATTCTGGAAGCACGCTTGTAGACTGAAGAACTTCACCGCG<br>GCCGTTCAAAGATGTTGCCAGATGAAGGATAGTGCCAAGACCCTGGTGTCG<br>GACTGTTTCCTGGAGTTCTACTCTGAGTTCATGATGGGCTATAGAGTGACTC<br>CTGCCGTGATCAAGTTTATGTTCACCGGCCTGATAAACTCCAGTGTGACAA<br>GCCCTCAGTCTCTGATGCAGGCCTGTCAGGTGAGCAGCCAGCAAGCTATGT<br>ACAACAGCGTCCCTCTGATCACCAATACCGCCTTCACCCTGCTGCGGCAGC<br>AAATCTTCTTCAATCACGTGGAAGATTTTATCAGACGGTACGGCATCCTGAC<br>CCTGGGCACCCTGTCACCTTTCGGCAGACTGTTCGTGCCTACCTACAGCGG<br>CCTGGTGTCTAGCGCCGTGGCCCTGGAGGACGCCGAGGTGATCGCCAGGG<br>CCGCCCAGACCCTGCACATGAACTCCGTCTCTATCCAAAGCTCTAGCCTGA<br>CAACCCTGGATTCACTGGGCCGGAGCAGAACCTCTAGCACAGTAGAAGAT<br>AGCTCTAGCGTGTCTGATACCACAGTCGCGAGCCACGATTCTGGCAGCAGT<br>AGCAGCTCCTTCTCTTTCGAACTGAATAGACCACTGAGCGAGACCGAGCTG<br>CAGTTCATCAAGGCCCTAAACAGCCTGAAATCCACACAGGCTTGTGAGGTG<br>ATTCAGAATCGTATCACAGGCCTGTACTGCAATTCGAATGAGGGCCCTCTGG<br>ATAGACACAACGTTATCTACTCCTCCCGGATGGCCGATAGTTGCGACTGGCT

	GCGGGACGGCAAGCGGAGAGGCAACCTGGAGCTGGCCAACAGAATCCAG<br>TCTGTGCTGTGCGTGCTAATAGCTGGATACTACAGATCTTTCGGCGGCGAGG<br>GCACAGAAAAGCAGGTGAAGGCCAGTCTGAATCGAGATGACAACAAGATC<br>ATCGAAGATCCTATGATTCAGCTGATCCCAGAGAAGCTGAGAAGGGAACTG<br>GAACGGCTGGGAGTGAGCAGAATGGAGGTGGACGAGCTGATGCCTAGCAT<br>CTCTCCTGACGACACCCTCGCTCAACTGGTTGCCAAAAAGCTGATTAGCCT<br>GAATGTGTCTACCGAAGAGTACTCTGCCGAGGTGAGCCGGCTGAAACAGA<br>CCCTGACAGCCAGAAACGTGTTGCACGGCCTTGCTGGAGGCATCAAGGAG<br>CTGTCCCTGCCTATCTACACCATTTTCATGAAATCTTACTTCTTTAAGGATAA<br>CGTGTTTCTGAGCCTGACAGACAGATGGTCCACCAAGCATTCTACCAACTA<br>CAGAGACTCCTGCGGCAAACAGCTGACCGGAAGAATCATCACCAAGTACA<br>CCCACTGGCTGGACACCTTCTTATCCTGCTCAGTTTCATTCAACCGGCACAC<br>CACCGTGAAAGAGCCTAGCCTGTTTAACCCTAACATCCGGTGCGTGAACCT<br>GATAACATTTGAAGATGGCCTGAGAGAACTAAGTGTGATTCAGAGCCACCT<br>CAAGGTCTTCGAGAACGAGTTCACAAACCTTAACCTGCAGTTTTCAGACCC<br>TAACAGGCAGAAACTGCGCATTGTGGAAAGCCGGCCTGCAGAAAGCGAAC<br>TTGAAGCGAACAGAGCTGTTATCGTTAAGACAAAGCTGTTCTCTGCCACAG<br>AGCAGGTTAGACTGAGCAACAACCCCGCCGTAGTGATGGGCTACCTGTTGG<br>ACGAGAGCGCCATCAGCGAGGTGAAGCCCACCAAGGTGGACTTTAGCAAT<br>CTGCTGAAGGACCGGTTTAAGATCATGCAATTCTTCCCTAGCGTGTTCACCC<br>TTATCAAAATGCTGGCCGACGAGTCCACCGACTCTGAAAAGAATGGCCTCT<br>CCCCCGACCTTCAGCAGGTGGCCAGATACAGCAATCACCTGACCCTGCTCT<br>CCAGAATGATCCAGCAGGCCAAGCCTACGGTGACAGTCTTCTATATGCTGA<br>AAGGAAACCTGATGAACACCGAACCCACTGTGGCTGAGCTGGTGAGCTAC<br>GGCATCAAGGAGGGCAGATTCTACAGATTGAGCGATACAGGTATCGACGCC<br>AGCACCTACAGCGTGAAATACTGGAAGATCCTGCATTGCATCAGCGCCATT<br>GGATGCCTGCCCCTGAGCCAGGCCGACAAGAGCAGCCTGCTGATGTCTTTT<br>CTTAATTGGCGGGTGAACATGGACATCAGAGCTAGCGACTGTCCTCTGTCC<br>AACCACGAGGCCTCTATACTGTCAGAGTTTGATGGACAGGTTATCGCTAAC<br>ATCTTGGCGAGCGAACTGAGCTCAGTGAAGCGGGACAGCGAAAGAGAAG<br>GCCTGACCGACCTGCTGGACTACTTGAACTCACCTACAGAACTGCTGAAGA<br>AAAAGCCCTACCTGGGGACAACTTGCAAGTTTAATACCTGGGGAGATTCCA<br>ACAGAAGCGGCAAGTTCACATACTCCTCAAGAAGCGGCGAGTCTATCGGG<br>ATCTTTATTGCCGGCAAGCTGCACATCCACCTGTCTAGCGAGTCTATCGCCC<br>TCCTGTGTGAAACCGAACGGCAGGTACTGAGCTGGATGAGCAAGAGACGG<br>ACCGAGGTGATCACAAAGGAACAGCACCAGCTGTTCCTGAGCCTGCTCCC<br>TCAGTCCCACGAGTGCCTGCAGAAGCACAAAGATGGCTCTGCCCTGAGCG<br>TGATCCCCGACGGCAGCAATCCTAGACTGCTGAAGTTCGTGCCTCTGAAGA<br>AAGGCCTCGCTGTGGTGAAGATCAAGAAGCAAATCCTGACAGTGAAGAAG<br>CAGGTGGTGTTCGACGCCGAGTCTGAGCCTAGACTGCAGTGGGGCCACGG<br>CTGCCTGAGCATCGTGTACGACGAGACCGACACCCAGACCACTTACCACG<br>AGAACCTGCTGAAGGTGAAGCAGCTGGTGGATTGCAGCACAGATAGAAAG<br>AAGCTGCTGCCTCAGAGCGTGTTCTCCGACAGCAAGGTGGTGCTGAGCCG<br>GATCAAATTCAAGACTGAGCTGCTGCTGAACAGCCTGACGCTGCTGCACTG

	CTTCCTGAAACACGCCCCCTCTGATGCTATAATGGAAGTGGAAAGTAAGTC<br>GAGCCTGCTGCATAAGTTCCTGAAGCTGGGCGGCGTGAGACAGAGAAACA<br>CCGAAGTTCTGCTCCGGGAGAAAATGAACAAGGTGGTGATCAAGGACAAT<br>CTGGAACAGGGCATGGAGGAGGAGATCGAGTTTTGTAACAACTTGACTAA<br>AACCGTGAGCGAAAACCCTCTGCCTCTGTCTTGTTGGTCTGAGGTGCAGAA<br>CTACATCGAGGACATCGGATTCAATAACGTGCTGGTGAACATTGATAGAAA<br>CACTGTGAAATCCGAGCTCCTGTGGAAATTCACGCTCGACACAAATGTCAG<br>CACCACCAGCACCATCAAAGACGTGCGTACCCTGGTTTCTTACGTGAGCAC<br>AGAGACAATCCCCAAGTTCCTGCTGGCCTTCCTGCTTTATGAGGAAGTGCT<br>CATGAACCTGATCTCTCAGTGTAAGGCCGTGAAGGAGCTTATTAACAGCAC<br>AGGCCTGTCCGACTTGGAACTGGAGAGCCTGCTGACCCTGTGTGCTTTTTA<br>CTTCCAGTCAGAATGTTCCAAGCGGGACGGTCCTAGATGCAGCTTCGCTGC<br>CTTGCTGTCCCTGGTGCACGAGGACTGGCAGAAAATCGGCAAGAACATCC<br>TCGTGCGAGCCAACAACGAGCTGGGCGACGTGTCCTTGAAGGTGAACATC<br>GTTTTGGTGCCCCTGAAGGATATGAGCAAACCTAAGCACGAGAGAGTGGT<br>GATCGCTAGAAGAAGCCTGAACCACGCTCTGTCACTAATGTTCCTGGACGA<br>GATGTCTCTTCCTGAGCTGAAGAGCCTCAGTCTGAACTGCAAGATGGGCAG<br>CCTGGAAGGCCAGGAGTGCTTCGAGTTCACCATCCTGAAGGACAATTCTAC<br>CAGACTGGACTATAACAAGCTGATCGACCACTGTGTGGACATGGAAAAGA<br>AGAGAGATGCCGTACGCGCCGTCGAGGACCTGATCCTGATGCTAACCGGCA<br>AGGCCGTGAAGCCCAGCAATGTGACCCAGATCGCCAGAGAGGATGAGCAG<br>TGCCAGGAGCAGATCAGCCTTGATGACCTGATGGCCTCTGACACCGTGATC<br>GACCTGCCAGATAGAGAAGCCGAGGCCCTAAAGACAGGGAACCTGGGCTT<br>CAACTGGGACAGCGATTGGTCGCACCCTCAGTTCGAGAAGGGCGGCGGCT<br>CTGGCGGGGGCAGCGGCGGCAGCGCCTGGAGCCACCCCCAGTTTGAAAAG<br>TGA

Supplementary Table S3

Oligonucleotides used in this work.

Oligonucleotides	Sequence (5′-3′ direction)	Description
5′-vRNA	UCUCAAAGAAAUCGUUCC	For cryo-EM sample preparation, the 5′ end was phosphorylated.<br>For BLI assays, the 5′ end was biotinylated.
5′-vRNA-U1A	ACUCAAAGAAAUCGUUCC	5′-Biotin
5′-vRNA-U1G	GCUCAAAGAAAUCGUUCC	5′-Biotin
5′-vRNA-U1C	CCUCAAAGAAAUCGUUCC	5′-Biotin
5′-vRNA-C2G	UGUCAAAGAAAUCGUUCC	5′-Biotin
5′-vRNA-C2A	UAUCAAAGAAAUCGUUCC	5′-Biotin
5′-vRNA-C2U	UUUCAAAGAAAUCGUUCC	5′-Biotin
5′-vRNA-U3A	UCACAAAGAAAUCGUUCC	5′-Biotin
5′-vRNA-U3G	UCGCAAAGAAAUCGUUCC	5′ ′-Biotin

5′-vRNA-U3C	UCCCAAAGAAAUCGUUCC	5′-Biotin
5′-vRNA-C4G	UCUGAAAGAAAUCGUUCC	5′-Biotin
5′-vRNA-C4A	UCUAAAAGAAAUCGUUCC	5′-Biotin
5′-vRNA-C4U	UCUUAAAGAAAUCGUUCC	5′-Biotin
5′-vRNA	UCUCAAAGAUAUAGCUGCGCGC	For ^{32}P-labelled radionucleotide extension assay
3′-vRNA	GCGCGCUGAUAUCUUUGCUA	For ^{32}P-labelled radionucleotide extension assay
